# Process Optimization and Performance Evaluation of CuCrZr Alloy Deposited by Blue–Near-Infrared Hybrid Laser Directed Energy Deposition

**DOI:** 10.3390/ma19214488

**Published:** 2026-10-24

**Authors:** Xuantong Liu, Xiaosong Zhou, Sen Yang, Quan Wu, Yongsheng Yu, Zejing Zhou, Huagui Zhang, Can Wang, Xue Qi

**Affiliations:** School of Mechanical and Electrical Engineering, Guizhou Normal University, Guiyang 550025, Chinasyang@gznu.edu.cn (S.Y.);

**Keywords:** blue–near-infrared hybrid laser, directed energy deposition, CuCrZr alloy, process parameter optimization, dilution ratio, tribological performance

## Abstract

**Highlights:**

- Hybrid blue–near-infrared laser directed energy deposition enabled stable CuCrZr deposition on T2 pure copper.
- Optimized processing achieved dilution near 15%, complete deposit cross-sections, and continuous interfaces.
- Compared with T2 pure copper, mean microhardness increased by 15.90% and specific wear rate decreased by 49.9%.

**Abstract:**

The high thermal conductivity and low initial near-infrared laser absorptivity of T2 pure copper hinder stable deposition of compositionally similar CuCrZr alloys. This study deposited CuCrZr on T2 copper using hybrid blue and near-infrared laser directed energy deposition, targeting 15% dilution. Single-factor experiments established the process window; Taguchi orthogonal experiments, signal-to-noise ratio analysis, and analysis of variance identified optimized parameters. Independent replicates and microstructural and performance characterization verified the optimization. FLOW-3D simulations supported analysis of deposit formation and interfacial fusion. Scanning speed and hatching distance were the main factors affecting dilution within the investigated window. The optimized near-infrared laser power, powder feed rate, scanning speed, and hatching distance were 2700 W, 12 g/min, 18 mm/s, and 0.7 mm, respectively. Validation experiments yielded 14.81% mean dilution, with complete deposit cross-sections and continuous interfaces. CT measurements indicated approximately 99.99% relative density in specimens deposited using these parameters. Compared with the substrate, mean microhardness increased by 15.90%, mean friction coefficient decreased by 20.5%, and wear volume and specific wear rate both decreased by 49.9%. This study provides a process basis for stable CuCrZr deposition and surface strengthening on pure copper.

## 1. Introduction

Copper and copper alloys have excellent electrical and thermal conductivity and stable performance in service. They are widely used in electrical and electronic applications, aerospace, and advanced equipment [1,2,3]. T2 pure copper has high electrical and thermal conductivity. However, copper components still experience substantial surface wear during frictional contact and interaction with particles. This wear produces grooves, microcracks, and material loss, indicating a need to improve surface wear resistance [2,4]. Laser directed energy deposition (L-DED) enables localized material addition and metallurgical bonding on substrate surfaces. It provides a feasible approach to modifying the surface properties of copper and copper alloys [5]. However, research on laser cladding of copper and copper alloy surfaces remains limited. In particular, the stable deposition of compositionally similar copper alloys on pure copper substrates has received little systematic investigation [6].

Previous studies have improved substrate hardness, wear resistance, and corrosion resistance by depositing various coatings on copper and copper alloys [6]. These include Ni-based, Co-based, Fe-based, and high-entropy alloy coatings, as well as particle-reinforced composite coatings. Zhang et al. [7] deposited Ni-based alloy coatings on pure copper using CO_2_ laser cladding with coaxial powder feeding. Without substrate preheating, continuous coatings were difficult to obtain even at an areal energy density of 1600 J/mm^2^. After preheating the substrate to 300 °C, continuous coatings with good bonding were obtained at an areal energy density of 256 J/mm^2^. For copper deposition, Asano et al. [8] compared blue and near-infrared lasers for depositing pure copper on 304 stainless steel substrates. With identical spot diameters and scanning speeds, the near-infrared laser produced continuous tracks at an areal energy density of 85.0 J/mm^2^. The blue laser achieved continuous deposition at an areal energy density of 54.4 J/mm^2^. Under these experimental conditions, blue laser irradiation achieved continuous copper deposition at a lower areal energy density. Copper has low initial absorptivity for near-infrared laser light, so a large proportion of the incident energy is reflected from its surface. This reflection limits the effective use of laser energy. Its high thermal conductivity also rapidly dissipates absorbed heat into the substrate, hindering local heat accumulation and stable molten pool formation [1,2]. Improving energy coupling between copper and the laser is consequently important. Blue laser irradiation provides substantially higher melting efficiency for pure copper than near-infrared laser irradiation [9]. Liu et al. [10] fabricated pure copper blocks using blue laser L-DED at an areal energy density of approximately 39.0 J/mm^2^. The blocks achieved a relative density of 99.6%, demonstrating the feasibility of blue laser deposition. However, larger specimens showed a lower relative density under the same processing conditions. This decrease was mainly associated with greater heat dissipation, insufficient melting, and inadequate interlayer fusion. Thus, copper’s high absorptivity for blue laser light does not eliminate the effects of rapid heat dissipation. At a given output power, blue lasers may still be unable to meet the deposition requirements of larger pure copper components.

The output power of individual blue semiconductor laser units is limited. Blue laser systems therefore increase their total output power through beam combining, although limitations remain in beam brightness and energy concentration [11]. Once the equipment’s maximum blue laser output is reached, adjusting blue laser power alone cannot further extend the total heat input range. Near-infrared laser irradiation can supplement the heat input while retaining the absorption advantage of blue laser irradiation. Adjusting the near-infrared laser power then extends the controllable heat input range, forming a blue–near-infrared hybrid laser processing approach. Previous studies have shown that hybrid laser sources can alter energy absorption and molten pool dynamics during copper processing. Such sources have also been used for copper welding [12,13,14]. Wei et al. [15] deposited pure copper on stainless steel substrates using coaxial blue–near-infrared hybrid lasers and investigated molten pool behavior under the hybrid heat source. Tan et al. [16] used hybrid lasers to prepare CoCrFeNi high-entropy alloy coatings on copper alloy surfaces. However, few studies have examined blue–near-infrared hybrid laser deposition of copper and copper alloys. These two studies involved copper feedstock and copper alloy substrates, respectively, but neither addressed Cu-based alloy deposition on pure copper substrates. For CuCrZr deposition on T2 pure copper, the effects of process parameters still require investigation under the specific material and processing conditions. These parameters include near-infrared laser power, powder feed rate, scanning speed, and hatching distance. Their effects on deposit formation, interfacial fusion, and dilution ratio must be examined. Here, the dilution ratio characterizes the extent to which substrate material melts into the deposited layer during deposition. The microhardness and wear resistance of the resulting deposited layer must then be evaluated.

This study used blue–near-infrared hybrid laser L-DED to investigate the feasibility of CuCrZr deposition on T2 pure copper and its surface strengthening effects. With blue laser power fixed, single-factor experiments examined how near-infrared laser power, powder feed rate, scanning speed, and hatching distance affected deposit formation and interfacial fusion. These experiments established a process window for continuous and stable deposition. A Taguchi orthogonal experimental design was then used to evaluate the main effects of the process factors and select a suitable parameter combination. Its stability was verified through repeated deposition experiments. The microstructure, microhardness, and tribological performance of the deposited layer produced using the optimized parameters were subsequently characterized. Comparisons with the T2 pure copper substrate evaluated the improvements in surface hardness and wear resistance provided by the CuCrZr deposited layer. The findings can guide stable deposition and surface strengthening using compositionally similar copper alloys on pure copper substrates.

## 2. Materials and Methods

### 2.1. Experimental Equipment and System

**Figure 1:**
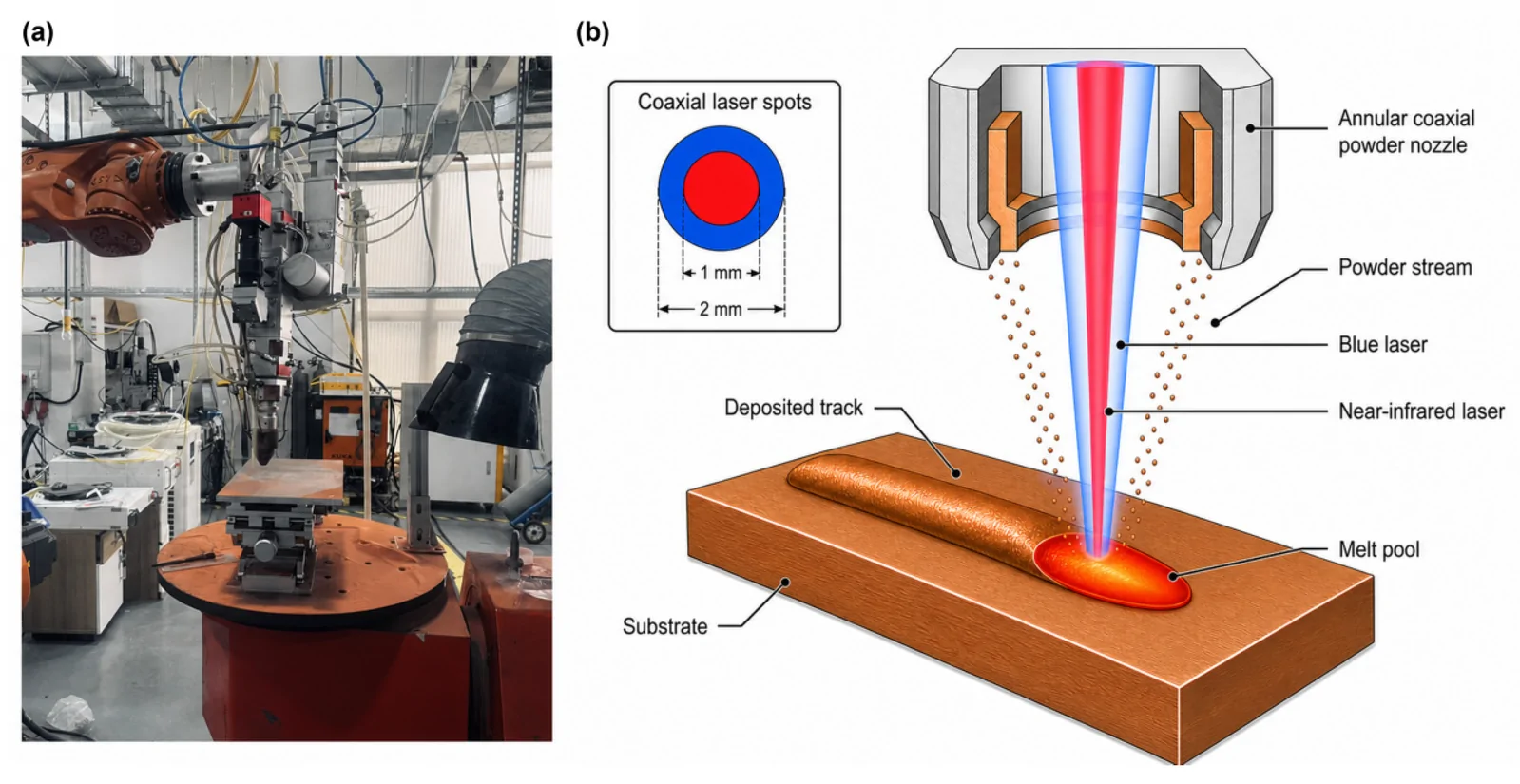
Blue–near-infrared hybrid laser L-DED system: (**a**) photograph of the experimental system; (**b**) schematic of blue–near-infrared hybrid laser irradiation.

Copper alloy deposition experiments were conducted using a custom-built blue–near-infrared hybrid laser L-DED system, as shown in Figure 1. The system was developed by the Key & Core Technology Innovation Institute of the Greater Bay Area(Guangzhou, China). It mainly consisted of near-infrared and blue lasers, an annular coaxial powder feeding system, an industrial robot motion control system, and a process monitoring system. The near-infrared laser had a wavelength of 1080 ± 20 nm and a maximum output power of 3000 W. The blue laser had a wavelength of 455 ± 10 nm and a maximum output power of 1000 W. The two beams were coaxially superimposed and irradiated the substrate surface together. Their spot diameters were 1 mm for the near-infrared laser and 2 mm for the blue laser. Together, they formed a blue–near-infrared hybrid laser heat source.

During deposition, CuCrZr alloy powder was delivered to the laser interaction zone through an annular coaxial powder feeding nozzle. The powder melted under hybrid laser irradiation and formed a molten pool with the partially melted substrate. The experiments were conducted in air, with argon used as the powder carrier gas. The industrial robot motion control system controlled the trajectory and scanning speed of the deposition head. The near-infrared laser mainly provided high-power energy input. The blue laser improved initial energy coupling by taking advantage of copper’s higher absorptivity at shorter wavelengths. The near-infrared beam could be approximated as having a Gaussian intensity distribution, whereas the blue beam could be approximated as having a flat-top distribution. Their coaxial hybrid configuration enhanced laser energy absorption at the copper alloy surface and provided stable heat input during L-DED.

### 2.2. Experimental Materials

CuCrZr alloy powder produced by Zhongyuan Advanced Materials Technologies Co.(Ningbo, China) was used as the deposition material. A T2 pure copper plate measuring 135 mm × 100 mm × 10 mm was used as the substrate. The chemical compositions of the CuCrZr alloy powder and T2 pure copper substrate are listed in Table 1. Before deposition, the powder was dried in an oven at 80 °C for 12 h to reduce the effects of adsorbed moisture on powder feeding stability and deposition. After cooling, the powder was used for the subsequent L-DED experiments. The substrate surface was ground with sandpaper, cleaned with anhydrous ethanol, and dried before deposition to remove surface oxides and oil contamination.

**Figure 2:**
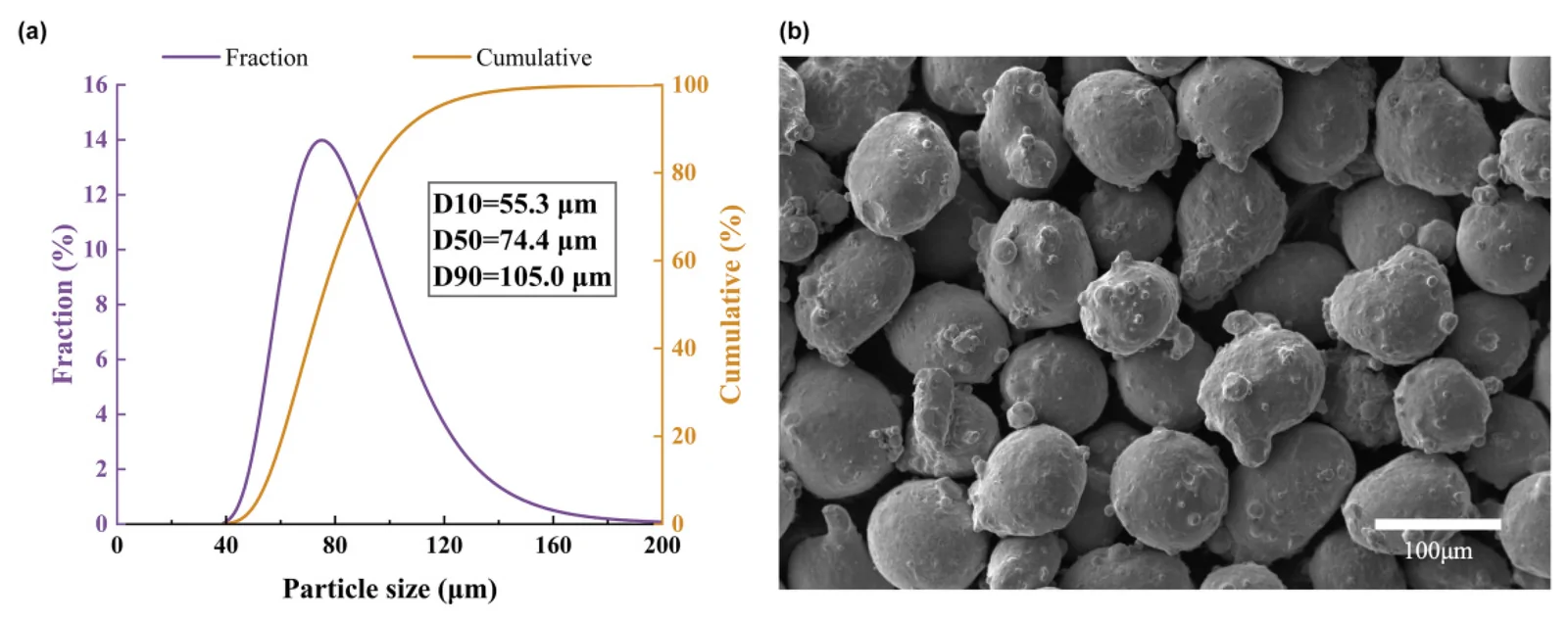
Particle size distribution and SEM morphology of the CuCrZr alloy powder: (**a**) particle size distribution; (**b**) SEM image.

The powder was produced by gas atomization, with a nominal particle size range of 53–105 μm specified by the manufacturer. The particle size distribution was measured by laser diffraction using a laser particle size analyzer (Mastersizer 3000, Malvern, UK). Powder morphology was examined using a scanning electron microscope (YB-300X, YINGBO, Shanghai, China). The results are shown in Figure 2a and Figure 2b, respectively. Particle size measurements gave D10, D50, and D90 values of 55.3, 74.4, and 105.0 μm, respectively. The SEM image showed predominantly near-spherical particles, with a small number of irregular particles and satellite particles attached to larger particles. These particle size and morphological characteristics met the basic requirements for subsequent coaxial powder feeding and L-DED experiments.

### 2.3. Experimental Design and Evaluation Criteria for Process Optimization

**Figure 3:**
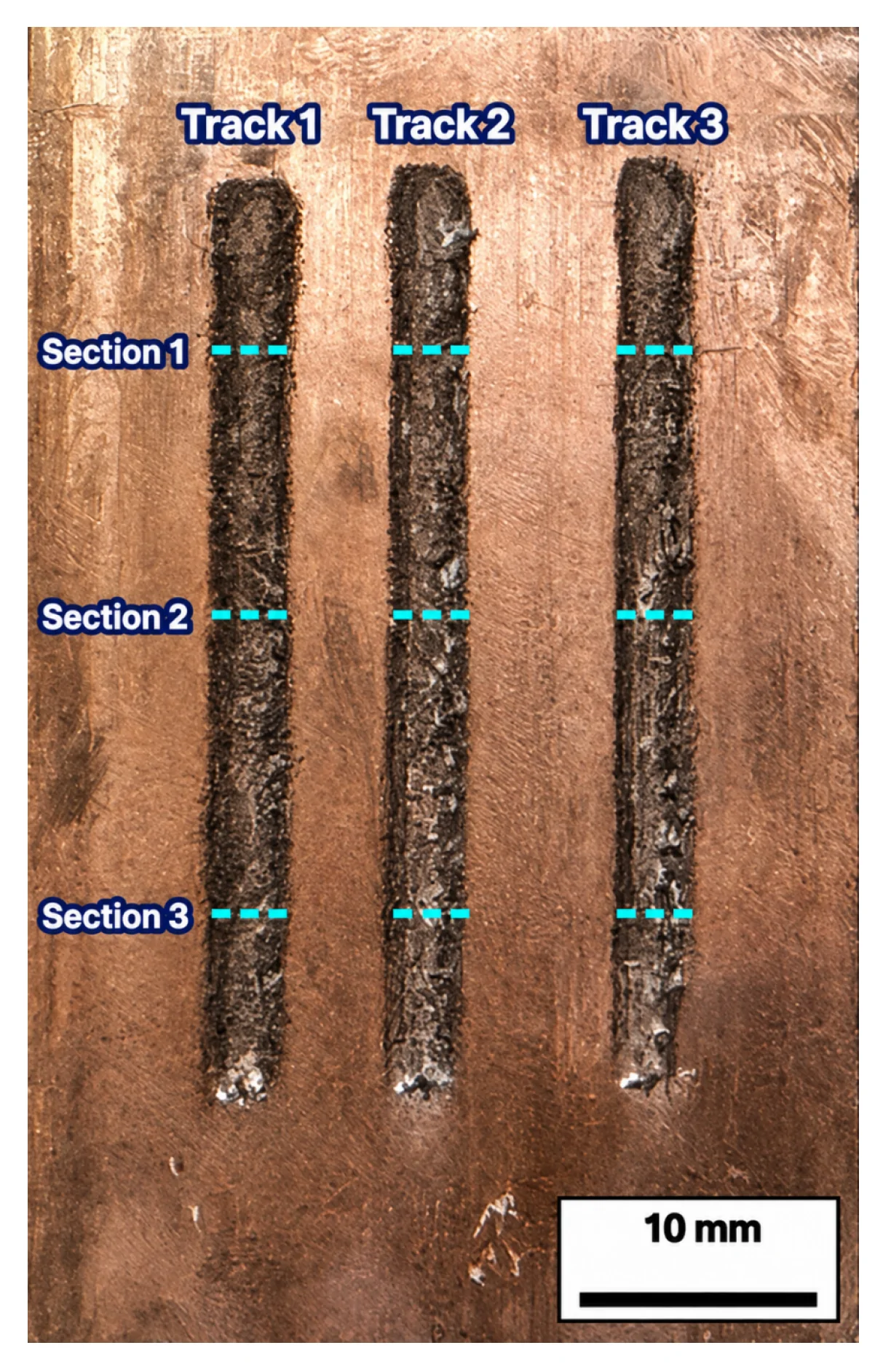
Schematic illustration of the sampling positions for metallographic cross-sections of the deposited tracks.

**Figure 4:**
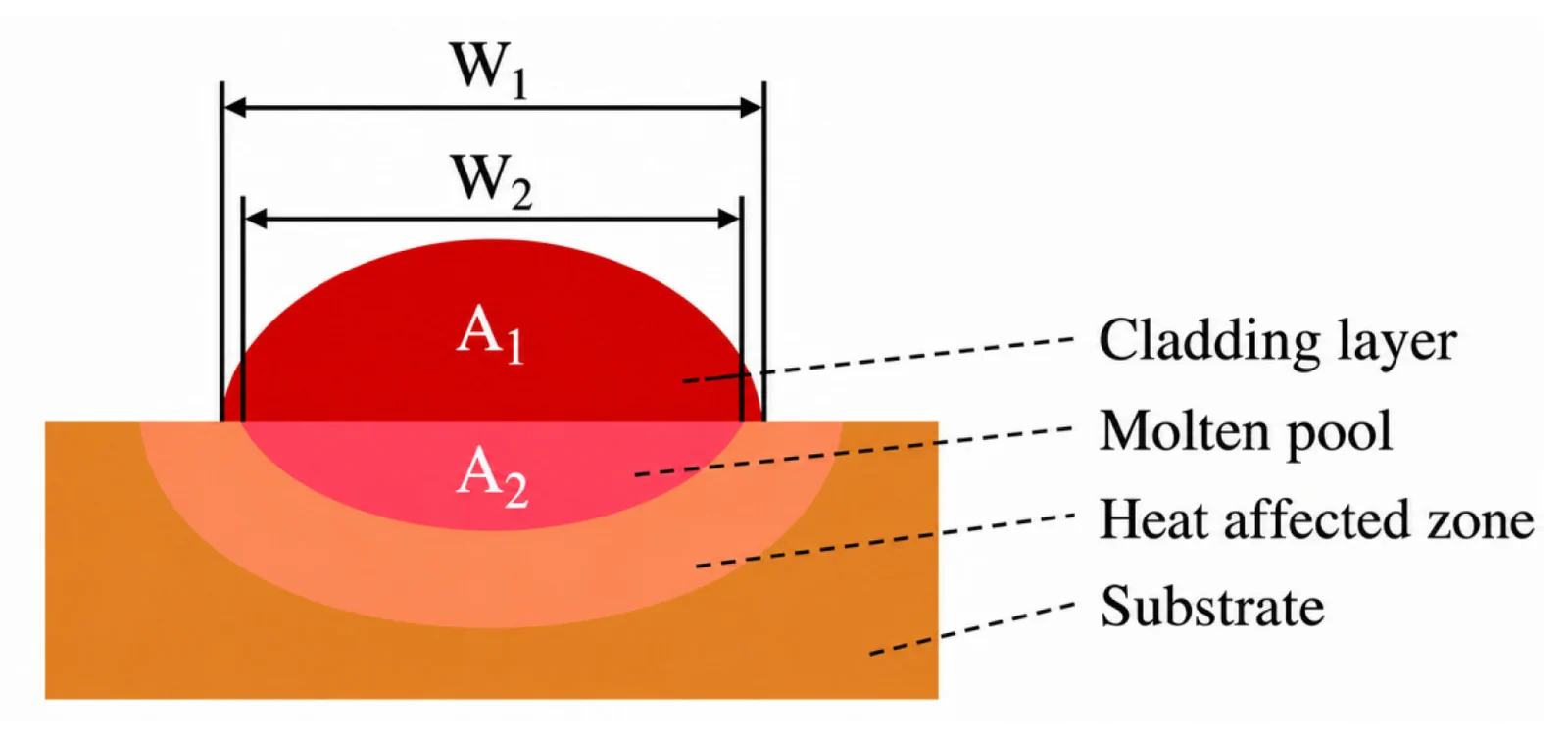
Schematic illustration of the measurement methods for dilution ratio and joining efficiency.

The dilution ratio was used as the primary evaluation metric for process optimization. Single-factor experiments first established the feasible process window for blue–near-infrared hybrid laser L-DED. A Taguchi orthogonal design was then used for multifactor parameter optimization. The blue laser power, powder carrier gas flow rate, and deposition length were fixed at 1000 W, 6.5 L/min, and 40 mm, respectively. The single-factor experiments examined the effects of near-infrared laser power, scanning speed, powder feed rate, and hatching distance on deposit formation and interfacial bonding. All single-factor experiments used single-track deposition, except those examining hatching distance. Near-infrared laser power ranged from 1500 to 2900 W in increments of 200 W. Scanning speed ranged from 6 to 22 mm/s in increments of 2 mm/s. Powder feed rate ranged from 7 to 15 g/min in increments of 1 g/min. Hatching distance ranged from 0.3 to 1.1 mm in increments of 0.1 mm. The parameter ranges are summarized in Table 2. To avoid errors caused by random variation, three tracks were independently fabricated for each set of process parameters. Three positions were selected along the length of each track, and the measurements were averaged. Cross-sectional specimens were prepared by wire cutting, and the specific sampling positions are shown in Figure 3. The specimens were sequentially mounted, ground, polished, and etched, and their cross-sectional morphologies were imaged using an optical microscope. The etchant contained 5 g FeCl_3_, 50 mL concentrated hydrochloric acid, and 100 mL H_2_O, and the etching time was 30 s. ImageJ (version 1.54t) was used for scale calibration and quantitative measurements of macroscopic track images and metallographic cross-sectional images. The measurement methods for dilution ratio and joining efficiency are illustrated in Figure 4. The macroscopic track width was measured from the track surface images. The effective fusion width was measured from the metallographic cross-sectional images at the corresponding positions. These images also provided the cross-sectional areas of the deposited layer above the substrate surface and the melted substrate region below it. Joining efficiency was calculated using Equation (1) [17], and dilution ratio was calculated using Equation (2) [18]:

(1) $$J=\frac{W_{2}}{W_{1}}\times 100\%$$

(2) $$\eta =\frac{A_{2}}{A_{1}+A_{2}}\times 100\%$$

Here, *J* is the joining efficiency, and *W*_1_ is the macroscopic track width at the corresponding position. *W*_2_ is the effective fusion width between the deposited layer and substrate in the metallographic cross-section, and *η* is the dilution ratio. *A*_1_ is the cross-sectional area of the deposited layer above the substrate surface. *A*_2_ is the cross-sectional area of the melted substrate region below the substrate surface. Engineering screening criteria for the single-factor experiments were established based on preliminary experiments and the cross-sectional morphologies of the deposits. Acceptable conditions required a continuous deposit surface, no obvious lack-of-fusion regions in the examined cross-sections, joining efficiency above 90%, and dilution ratio within 5–30%. These criteria excluded conditions with poor bonding or insufficient or excessive substrate melting. They also retained a broad parameter range for subsequent orthogonal optimization. Previous studies commonly recommended a dilution ratio of 10–20% for laser cladding [19,20,21]. The midpoint of this range, 15%, was therefore selected as the target dilution ratio. Based on the single-factor results, near-infrared laser power, scanning speed, hatching distance, and powder feed rate were selected for a four-factor, four-level Taguchi orthogonal design. Nominal-the-best signal-to-noise (S/N) ratio analysis was used to identify a parameter combination approaching the target dilution ratio. Analysis of variance (ANOVA) was then performed on the raw dilution ratio data from independent replicates in the orthogonal experiments. This analysis evaluated the statistical significance and percentage contribution of each process parameter’s main effect on dilution ratio. After optimization, the selected parameter combination was used to prepare deposited specimens for cross-sectional formation validation, microhardness testing, and tribological performance testing.

### 2.4. Microstructural and Defect Characterization and Performance Testing Methods

Cross-sectional morphologies and defect characteristics of deposits produced using different parameter combinations were examined with a high-resolution Schottky scanning electron microscope (SU3800SE Plus, Hitachi High-Tech Corporation, Tokyo, Japan). The instrument was equipped with an EBSD detector (eWARP, BrukerNano GmbH, Berlin, Germany). EBSD analysis was performed on the CuCrZr/T2 pure copper single-track deposited cross-section produced using the optimized parameters. Grain morphology, grain size, and grain boundary characteristics were examined in both the deposited layer and substrate. A multilayer, multitrack CuCrZr deposit produced using the optimized parameters was scanned with an X-ray microscope (nanoVoxel 3000, Sanying Precision Instruments Co., Ltd., Tianjin, China). The scanning voltage and current were 150 kV and 95 μA, respectively, and the voxel size was 5 μm. CT Pro 3D and Dragonfly were used for three-dimensional reconstruction and quantitative defect analysis.

**Figure 5:**
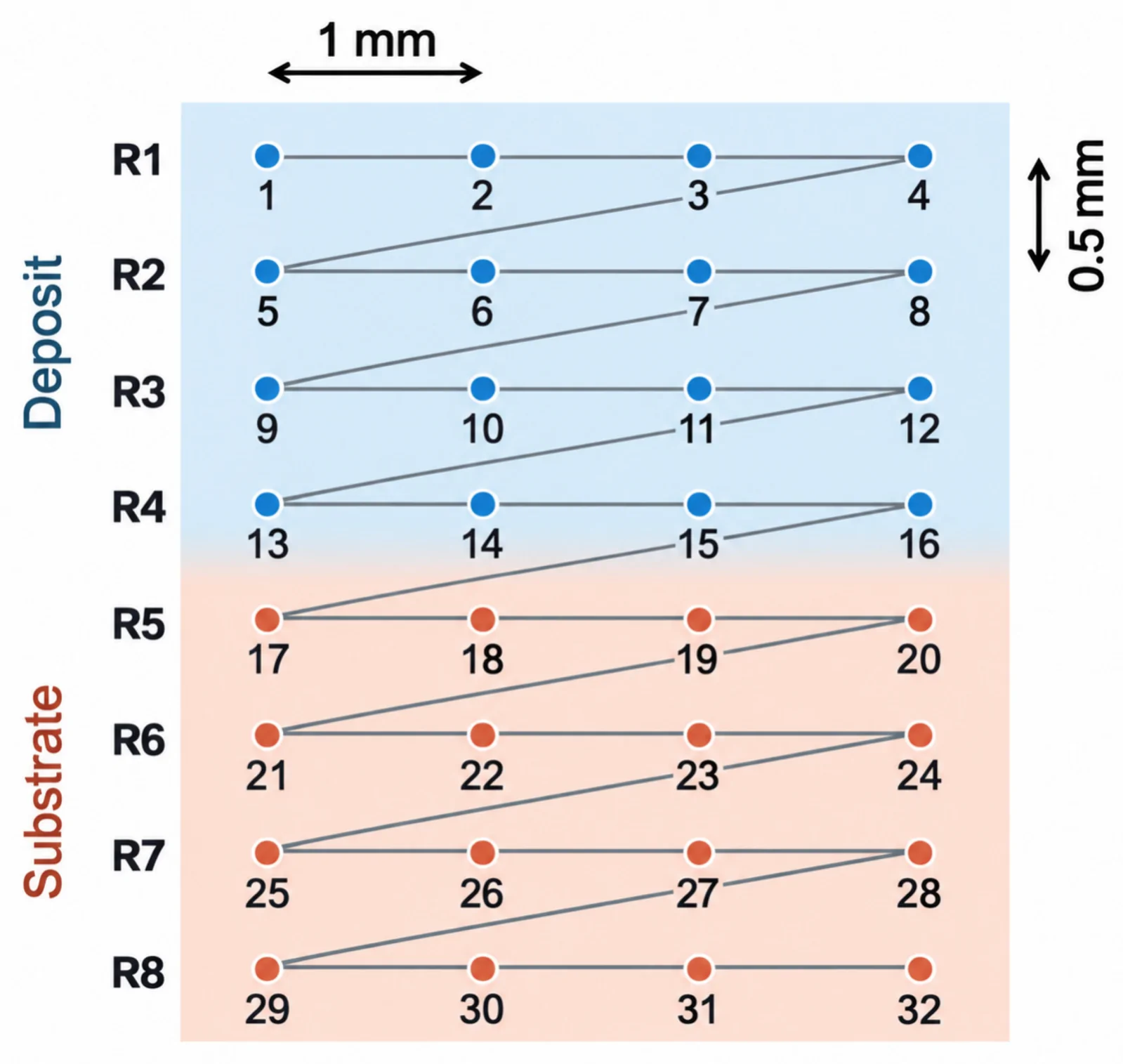
Schematic illustration of the microhardness measurement locations.

Microhardness and tribological tests were conducted to compare the CuCrZr deposited layer prepared using the optimized parameters with the T2 pure copper substrate. T2 pure copper substrate specimens were used as controls. Micro-Vickers hardness measurements were performed on a three-layer CuCrZr deposited specimen using a FALCON 500 Vickers hardness tester (INNOVATEST Europe BV, Maastricht, The Netherlands), with reference to ASTM E92-23 [22]. The applied load was 1.961 N (HV0.2), with a dwell time of 15 s. As shown in Figure 5, four measurement rows were arranged in each of the deposited and substrate regions of the specimen cross-section. Each row contained four measurement points, giving a total of 32 points. The vertical spacing between adjacent rows was 0.5 mm, and the horizontal spacing between adjacent points within each row was 1 mm. Points 1–16 were located in the deposited region, corresponding to rows R1–R4. Points 17–32 were located in the substrate region, corresponding to rows R5–R8. The mean and standard deviation of the four measurements in each row were calculated to characterize the hardness distribution and within-row variability.

Dry sliding friction and wear tests were performed on the CuCrZr deposited layer and T2 pure copper substrate using an MFT-5000 tribometer (Rtec-Instruments, Inc., San Jose, USA). The tests used a rotating ball-on-disc configuration, with reference to ASTM G99-23 [23]. The counterbody was an Al_2_O_3_ ceramic ball with a diameter of 10 mm. The normal load, sliding speed, and total sliding distance were 10 N, 0.15 m/s, and 150 m, respectively. The circular wear track radius was 20 mm, and all tests were conducted at room temperature. Three independent replicate tests were performed for each material. The coefficient of friction was recorded in real time as a function of sliding time. Values from the three independent tests were arithmetically averaged at matching sliding times to obtain the mean friction curves for the substrate and deposited layer.

After wear testing, the wear tracks were scanned using a 3D optical profilometer(SuperView W1, Chotest Technology Inc., Shenzhen, China) to obtain their three-dimensional topographies. Cross-sectional profiles perpendicular to the sliding direction were extracted from the three independent circular wear tracks, and their cross-sectional areas were calculated. The mean wear track cross-sectional area was calculated using Equation (3), and the wear volume of the circular track was calculated using Equation (4) [24]:

(3) $$\overset{-}{S}=\frac{S_{1}+S_{2}+S_{3}}{3}$$

(4) $$V=2\mathrm{\pi R}\overset{-}{S}$$

Here, *S*_1_, *S*_2_, and *S*_3_ are the cross-sectional areas of the three independent circular wear tracks, and $\overset{-}{S}$ is their mean cross-sectional area. *R* is the wear track radius, and *V* is the wear volume. The specific wear rate was calculated using Equation (5) [24]:

(5) $$K=\frac{V}{\mathrm{FL}}$$

Here, *K* is the specific wear rate, *V* is the wear volume, *F* is the normal load, and *L* is the total sliding distance. Wear volume and specific wear rate were also calculated separately from the cross-sectional area of each independent wear track. The replicate results were summarized as means and standard deviations. These values were used to quantitatively compare the wear resistance of the CuCrZr deposited layer and T2 pure copper substrate.

### 2.5. Numerical Simulation Methods

A numerical model of multitrack deposition with overlapping tracks was developed in FLOW-3D. The model supported analysis of molten pool thermal behavior, flow characteristics, and dilution development during blue–near-infrared hybrid laser L-DED. The simulations modeled multitrack deposition of CuCrZr alloy powder on a pure copper substrate. They accounted for particle motion, heating, melting, and interactions with the molten pool after particles entered the laser interaction zone. The optimized parameter combination described above was used in the simulations.

Molten pool flow and heat transfer were described using conservation equations for mass, momentum, and energy. The governing equations are given in Equations (6)–(8) [25]:

(6) $$\frac{\partial \rho }{\partial t}+\nabla \cdot \left(\rho \vec{v}\right)=0$$

(7) $$\frac{\partial \left(\rho \vec{v}\right)}{\partial t}+\nabla \cdot \left(\rho \vec{v}\vec{v}\right)=-\nabla p+\nabla \tau +\rho \vec{g}+{\vec{F}}_{\mathrm{sv}}$$

(8) $$\frac{\partial \left(\mathrm{\rho h}\right)}{\partial t}+\nabla \cdot \left(\mathrm{\rho h}\vec{v}\right)=\frac{\mathrm{Dp}}{\mathrm{Dt}}+\Phi +\nabla \cdot \left(k\nabla T\right)+Q$$

Here, ρ is the density; $\vec{v}$ is the fluid velocity vector; t is time; p is pressure; τ is the viscous stress tensor; $\vec{g}$ is gravitational acceleration; ${\vec{F}}_{\mathrm{sv}}$ is the volumetric force source term; h is enthalpy; k is thermal conductivity; T is temperature; Φ is viscous dissipation; Q is the external heat source term. ${\vec{F}}_{\mathrm{sv}}$ mainly includes surface tension, vapor recoil pressure, and Marangoni convection. These contributions describe forces and flow at the molten pool free surface.

The hybrid laser heat source was constructed by coaxially superimposing the near-infrared and blue laser heat sources. The total heat flux was the sum of their respective contributions. The near-infrared laser was represented by a Gaussian distribution, whereas the blue laser was represented by a flat-top distribution. The heat flux expressions are given in Equations (9)–(11) [26]:

*q* = *q_red_* + *q_blue_* (9)

(10) $$q_{\mathrm{red}}=\frac{2\eta_{\mathrm{red}}P_{\mathrm{red}}}{\pi R_{r}^{2}}\exp\left[-\frac{2\left((x-x_{0}-\mathrm{vt}{)}^{2}+(y-y_{0}{)}^{2}\right)}{R_{r}^{2}}\right]$$

(11) $$q_{\mathrm{blue}}=\frac{\eta_{\mathrm{blue}}P_{\mathrm{blue}}}{\pi R_{b}^{2}}\exp\left[-{\left(\frac{\sqrt{(x-x_{0}-\mathrm{vt}{)}^{2}+(y-y_{0}{)}^{2}}}{R_{b}}\right)}^{30}\right]$$

**Figure 6:**
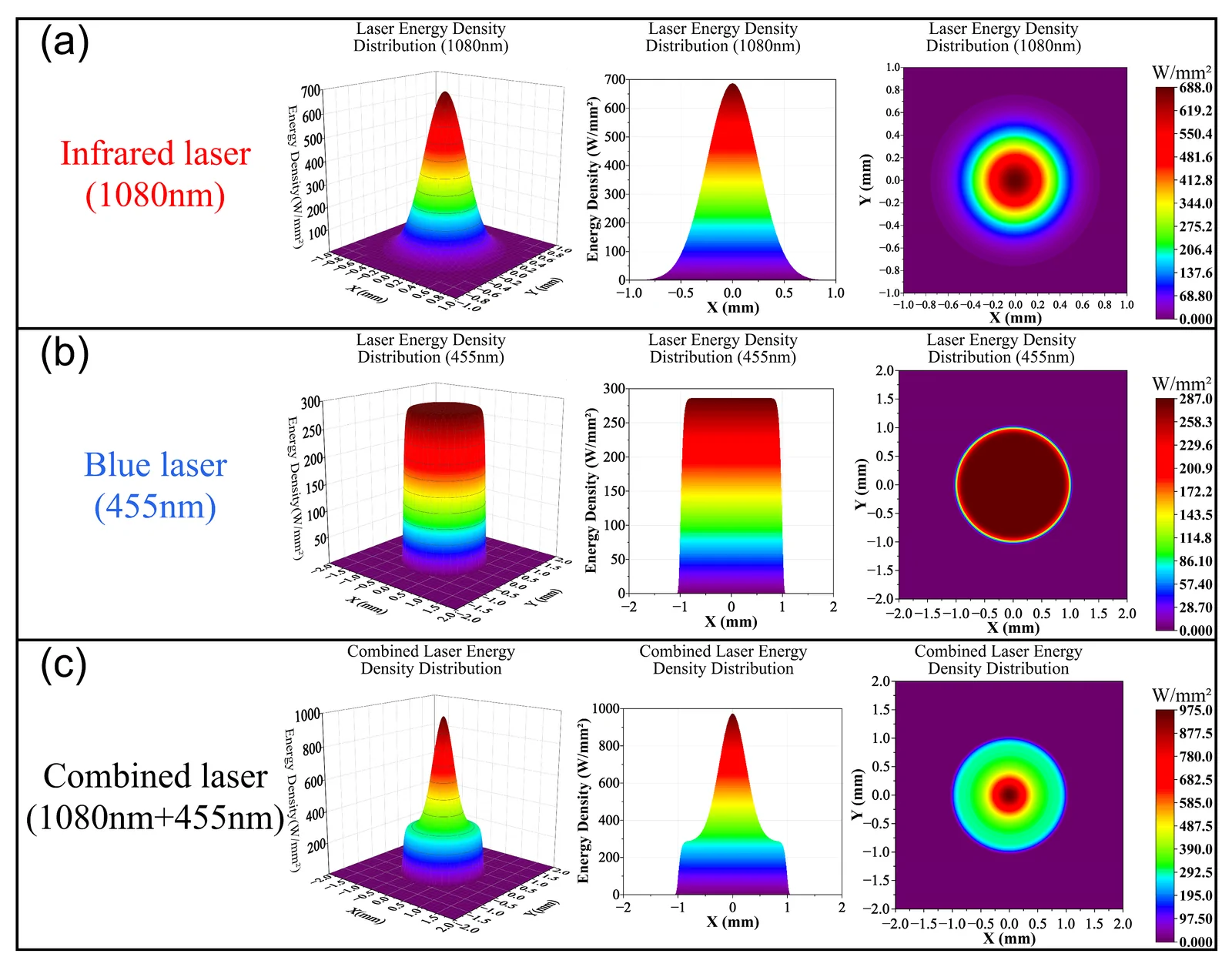
Energy distributions of the near-infrared, blue, and hybrid laser heat sources: (**a**) near-infrared Gaussian heat source; (**b**) blue flat-top heat source; (**c**) hybrid heat source.

Here, *q_red_* and *q_blue_* are the near-infrared and blue laser heat fluxes, respectively; *η_red_* and *η_blue_* are their respective absorptivities; *P_red_* and *P_blue_* are their respective laser powers; *R_r_* and *R_b_* are their respective spot radii; *x*_0_ and *y*_0_ specify the initial laser position; *v* is the scanning speed; *t* is the interaction time. The near-infrared and blue laser spot diameters were 1 and 2 mm, respectively. The optimized near-infrared power was used, and blue laser power was fixed at 1000 W. The energy distributions calculated using these heat source models are shown in Figure 6.

The powder was treated as a discrete particle phase. Particle motion, heating, and melting in the laser interaction zone were described with reference to published numerical models of laser metal deposition [27]. The particle equation of motion is given in Equation (12):

(12) $$\frac{du_{p}}{dt}=-\frac{1}{\rho_{p}}\nabla p+g+\beta_{d}(u-u’)\left|u-u’\right|\frac{\rho }{\rho_{p}}$$

Here, ***u****_p_* is the powder particle velocity; ***u*** is the surrounding fluid velocity; *ρ_p_* is the powder particle density; *ρ* is the surrounding fluid density; *p* is pressure; ***g*** is gravitational acceleration; *β_d_* is the drag coefficient. The particle velocity term is decomposed as follows:

[***u***’ = ***u****_p_* + ***u***_diff_] (13)

Here, ***u***_diff_ is the particle diffusion velocity. Particle heat transfer accounts for laser energy absorption, convective exchange with the surrounding fluid, and evaporative heat loss. The energy equation is given in Equation (14) [27]:

(14) $$m_{p}C_{p}\frac{dT_{p}}{dt}=Q_{p,\mathrm{abs}}+h_{c}A_{p}(T_{f}-T_{p})-{\dot{m}}_{v,p}L_{v}$$

Here, *m_p_* is the powder particle mass; *C_p_* is its specific heat capacity; *T_p_* is its temperature; *Q_p_*_,abs_ is the laser power actually absorbed by the particle; *h_c_* is the convective heat transfer coefficient; *A_p_* is particle surface area; *T_f_* is the surrounding fluid temperature; ${\dot{m}}_{v,p}$ is the particle evaporation mass rate; *L_v_* is the latent heat of vaporization. Laser energy absorbed by particles was calculated using ray tracing, accounting for particle shadowing, laser reflection, and energy attenuation [28]. Heated and melted particles entered the molten pool and contributed to deposit formation. Molten pool boundary conditions included convective, radiative, and evaporative heat losses. The effects of surface tension, vapor recoil pressure, and the Marangoni effect on free-surface flow were also considered. The simulations yielded temperature distributions, molten pool morphologies, and molten pool velocities during multitrack deposition.

## 3. Results

### 3.1. Single-Factor Experimental Results and Process Window Determination

**Figure 7:**
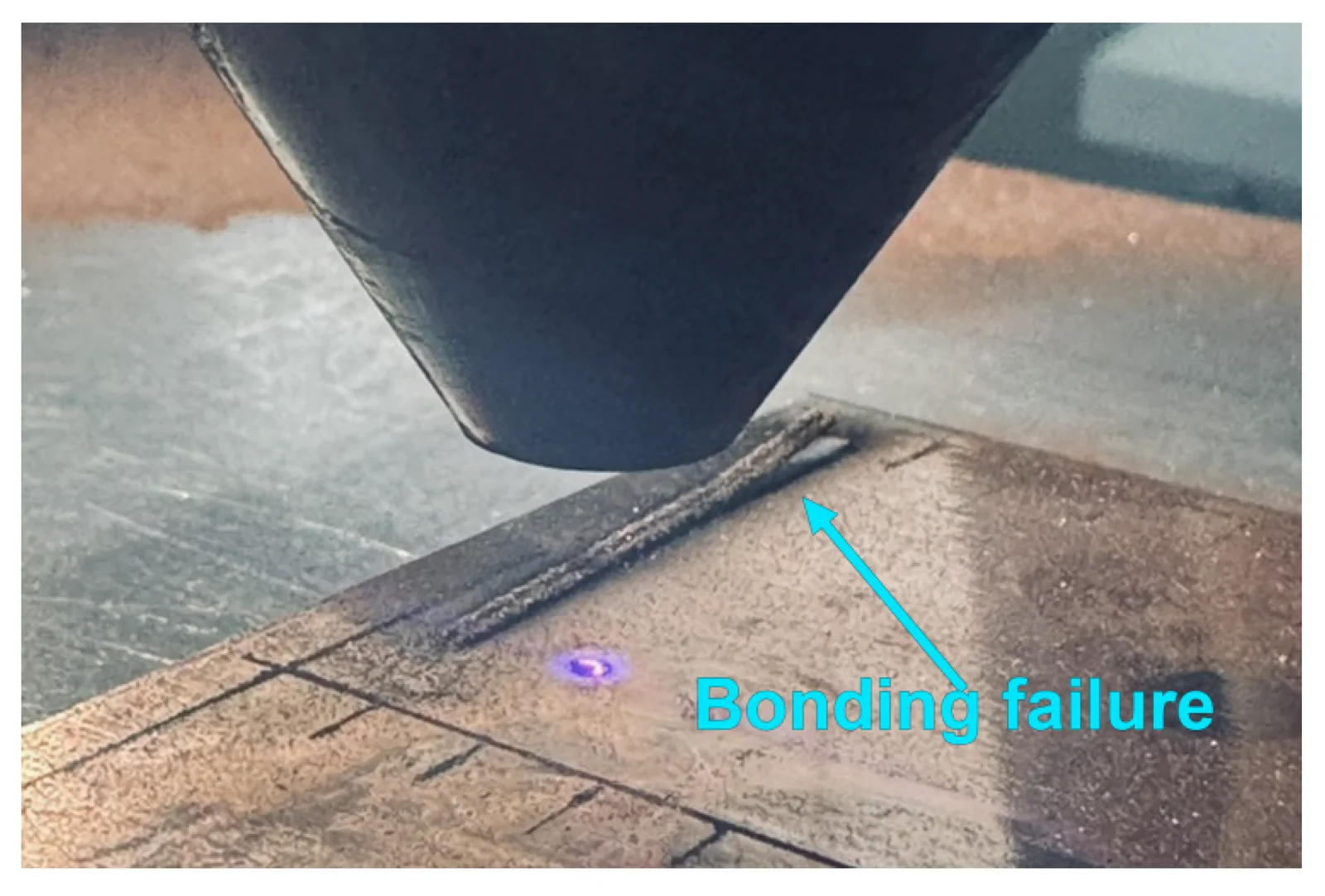
Macroscopic morphology of unstable CuCrZr deposition on a T2 pure copper substrate using an 800 W blue laser and a 2900 W near-infrared laser.

In preliminary trials, combining an 800 W blue laser with a 2900 W near-infrared laser failed to achieve stable CuCrZr deposition on T2 pure copper. The macroscopic morphology of the failed deposit is shown in Figure 7. Based on engineering experience provided by the equipment supplier and the preliminary results, blue laser power was increased to 1000 W. Stable track deposition was then achieved by combining this blue laser output with near-infrared irradiation.

Therefore, the blue laser power was fixed at 1000 W in subsequent experiments. Single-factor experiments on near-infrared laser power, powder feed rate, scanning speed, and hatching distance were conducted to establish a stable process window for blue–near-infrared hybrid laser L-DED. The usable range of each parameter was screened according to the criteria described in Section 2.3. This screening considered macroscopic deposit formation, fusion conditions in the examined cross-sections, joining efficiency, and dilution ratio. The experimental results and process window determination for each factor are presented below.

#### 3.1.1. Near-Infrared Laser Power

**Figure 8:**
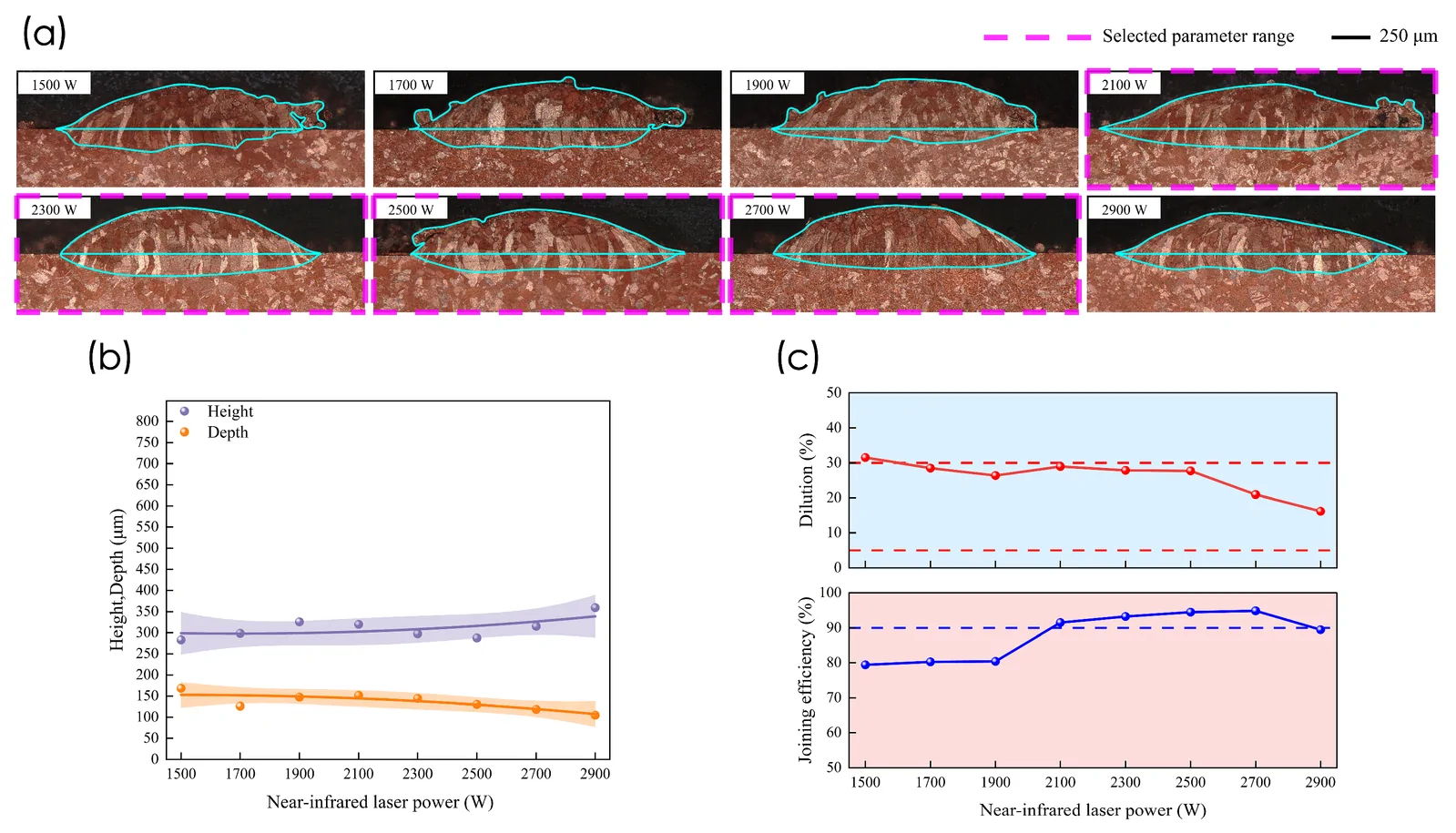
Cross-sectional morphologies and forming characteristics of the deposits at different near-infrared laser powers: (**a**) cross-sectional morphologies and selected parameter range; (**b**) deposit height and penetration depth; (**c**) indicate the dilution ratio screening limits of 5% and 30%, and the blue dashed line indicates the joining efficiency screening threshold of 90%.

Figure 8 shows the cross-sectional morphologies, geometric dimensions, dilution ratios, and joining efficiencies of the deposits at different near-infrared laser powers. As the power increased from 1500 to 2900 W, deposit height increased slightly overall, while penetration depth decreased slightly overall. The dilution ratio showed an overall decreasing trend with fluctuations as the power increased. It was slightly above 30% at 1500 W and approximately 26–29% within the range of 1700–2500 W. At 2700 and 2900 W, it decreased to approximately 21% and 16%, respectively. Joining efficiency was approximately 79–80% at 1500–1900 W, below the 90% screening threshold. It increased to above 90% at 2100 W and remained high within the range of 2300–2700 W. At 2900 W, joining efficiency decreased to approximately 89%, below the screening threshold. According to the criteria in Section 2.3, the dilution ratio at 1500 W exceeded the upper limit of 30%. Joining efficiencies at 1500–1900 W and 2900 W were below the 90% threshold. Therefore, 2100, 2300, 2500, and 2700 W were selected as the near-infrared laser power levels for the subsequent orthogonal experiments.

#### 3.1.2. Powder Feed Rate

**Figure 9:**
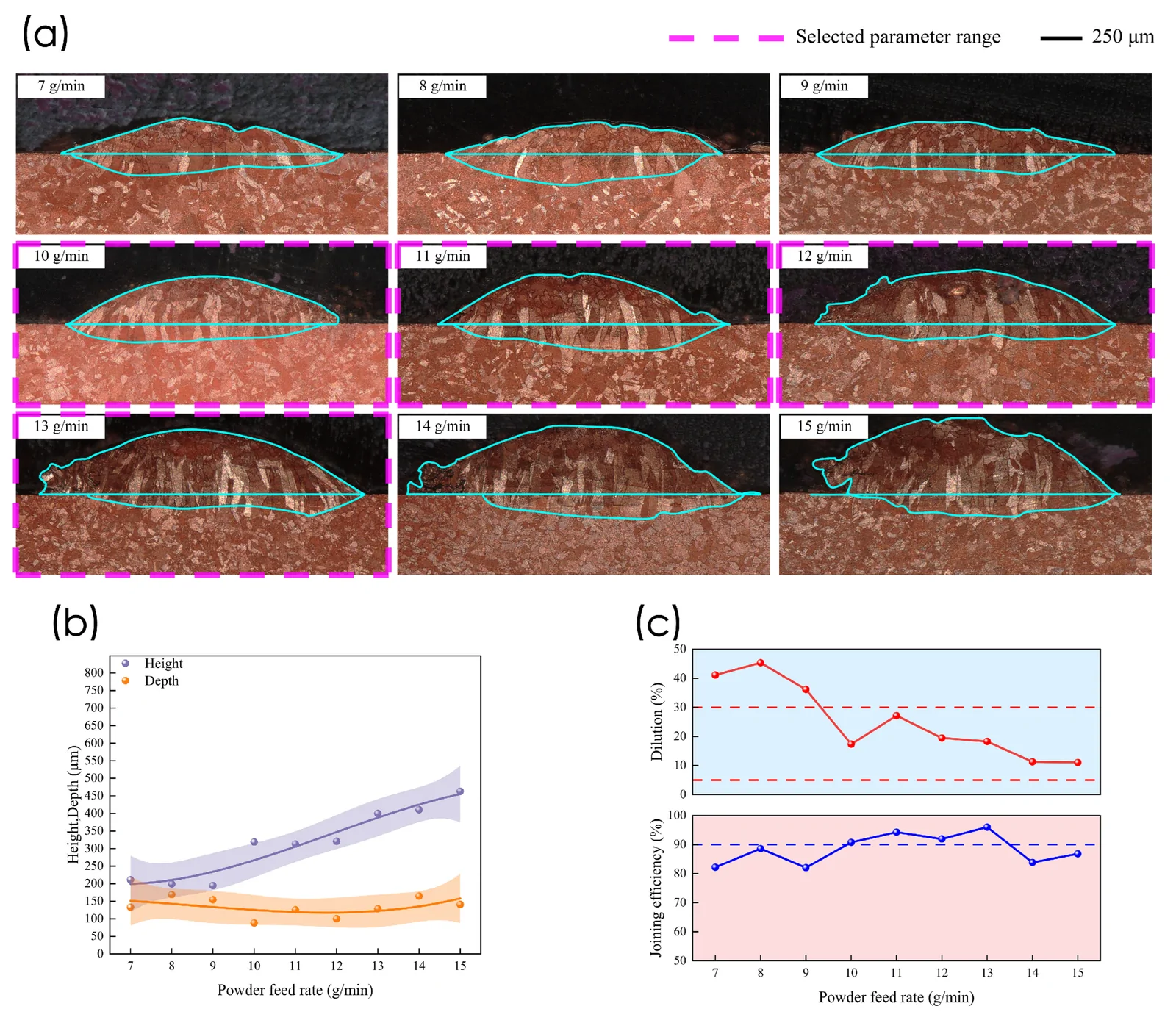
Cross-sectional morphologies and forming characteristics of the deposits at different powder feed rates: (**a**) cross-sectional morphologies and selected parameter range; (**b**) deposit height and penetration depth; (**c**) indicate the dilution ratio screening limits of 5% and 30%, and the blue dashed line indicates the joining efficiency screening threshold of 90%.

Figure 9 shows the cross-sectional morphologies, geometric dimensions, dilution ratios, and joining efficiencies of the deposits at different powder feed rates. As the powder feed rate increased from 7 to 15 g/min, deposit height increased overall. Penetration depth initially decreased and then increased slightly at higher powder feed rates. At 7–9 g/min, the dilution ratio was approximately 36–45%, exceeding the upper screening limit of 30%. At 10 g/min, the dilution ratio decreased to approximately 17%, while joining efficiency increased to above 90%. Within the range of 10–13 g/min, dilution ratios were approximately 17–27%, and joining efficiencies were all above 90%, satisfying the screening criteria. When the powder feed rate increased further to 14 and 15 g/min, dilution ratios remained low. However, joining efficiencies decreased to approximately 84% and 87%, respectively, below the 90% screening threshold. Therefore, 10, 11, 12, and 13 g/min were selected as the powder feed rate levels for the subsequent orthogonal experiments.

#### 3.1.3. Scanning Speed

**Figure 10:**
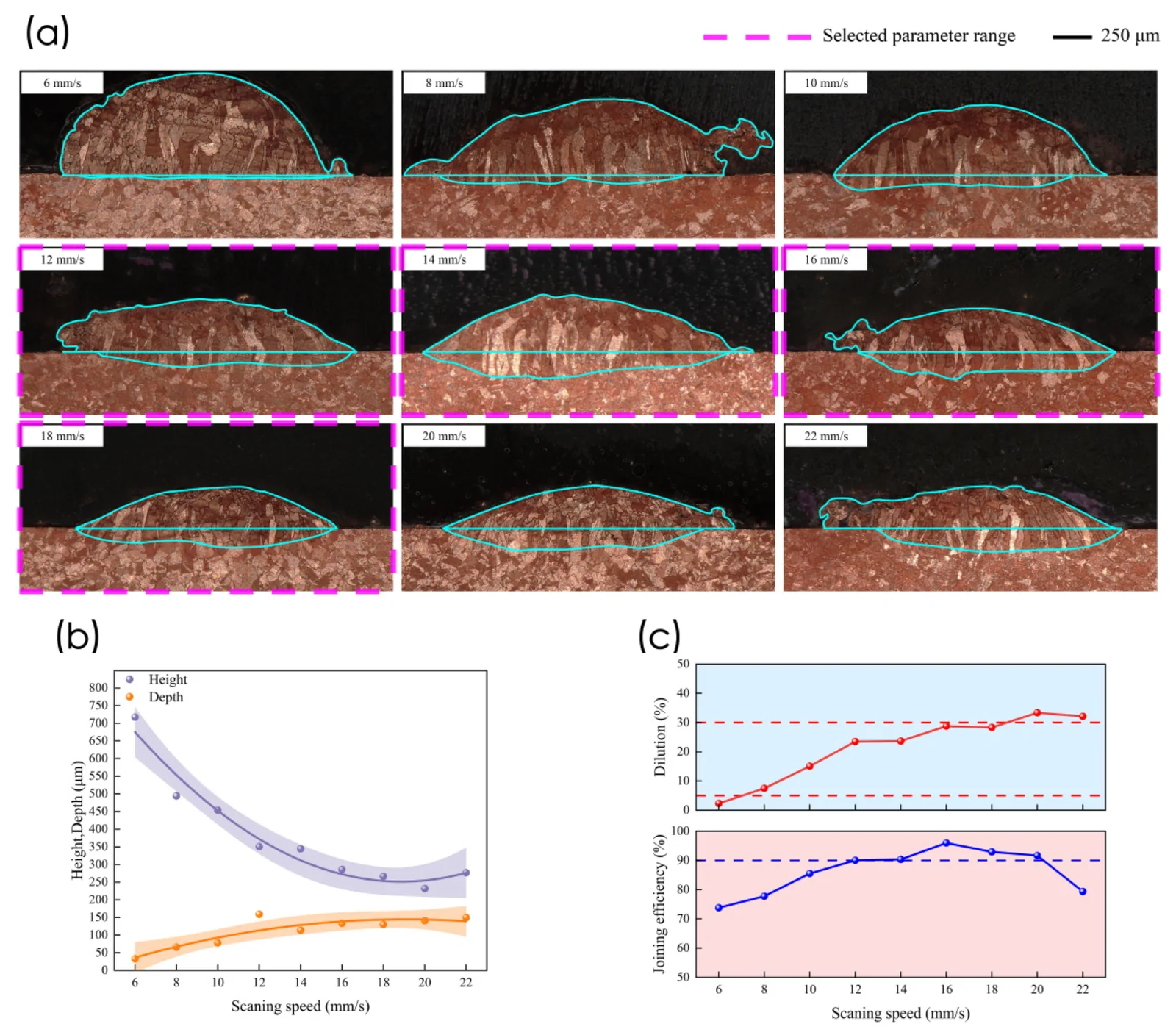
Cross-sectional morphologies and forming characteristics of the deposits at different scanning speeds: (**a**) cross-sectional morphologies and selected parameter range; (**b**) deposit height and penetration depth; (**c**) indicate the dilution ratio screening limits of 5% and 30%, and the blue dashed line indicates the joining efficiency screening threshold of 90%.

Figure 10 shows the cross-sectional morphologies, geometric dimensions, dilution ratios, and joining efficiencies of the deposits at different scanning speeds. As scanning speed increased from 6 to 22 mm/s, deposit height decreased overall. Within the range of 6–18 mm/s, it decreased from approximately 720 to 265 μm, with smaller changes at higher scanning speeds. Penetration depth increased overall but fluctuated across the tested scanning speeds. The dilution ratio increased overall, from approximately 2% at 6 mm/s to approximately 32–33% at 20–22 mm/s. At 6–10 mm/s, joining efficiency was below 90%, and the dilution ratio at 6 mm/s was also below the lower screening limit of 5%. At 12–18 mm/s, dilution ratios were approximately 23–29%, and joining efficiencies reached approximately 90% or higher, satisfying the screening criteria. At 20 mm/s, the dilution ratio exceeded 30%, so this condition failed the dilution ratio criterion despite a joining efficiency above 90%. At 22 mm/s, the dilution ratio remained above 30%, while joining efficiency decreased to approximately 79%. Therefore, 12, 14, 16, and 18 mm/s were selected as the scanning speed levels for the subsequent orthogonal experiments.

#### 3.1.4. Hatching Distance

**Figure 11:**
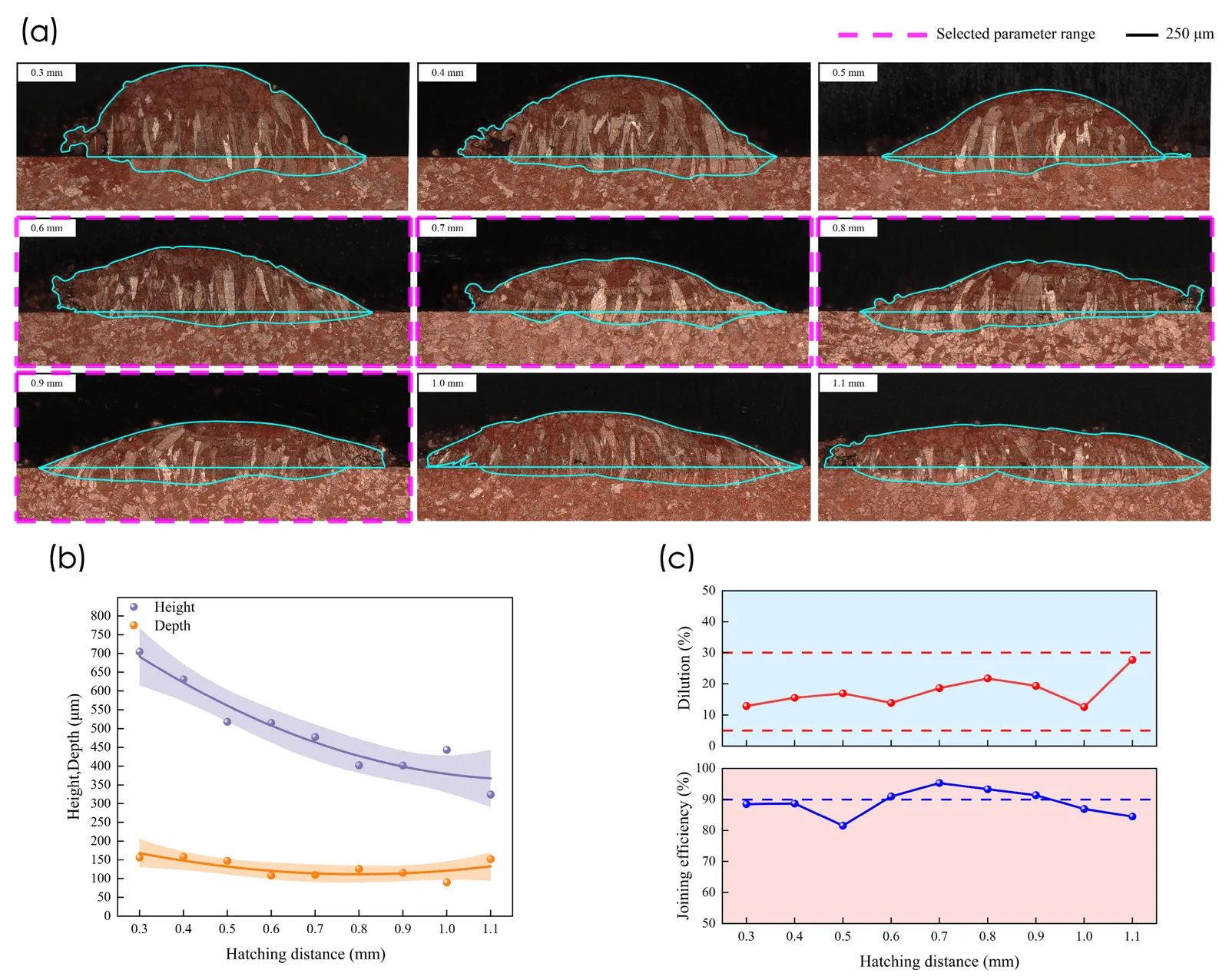
Cross-sectional morphologies and forming characteristics of the multi-track deposits at different hatching distances: (**a**) cross-sectional morphologies and selected parameter range; (**b**) deposit height and penetration depth; (**c**) indicate the dilution ratio screening limits of 5% and 30%, and the blue dashed line indicates the joining efficiency screening threshold of 90%.

Figure 11 shows the cross-sectional morphologies, geometric dimensions, dilution ratios, and joining efficiencies of the deposits at different hatching distances. As hatching distance increased from 0.3 to 1.1 mm, the multi-track deposit cross-sections generally changed from tall and narrow to wide and flat. Deposit height gradually decreased from approximately 700 to 325 μm, while penetration depth fluctuated within approximately 90–160 μm. Dilution ratios were approximately 12–28% across the tested hatching distances, all within the screening range of 5–30%. At 0.3–0.5 mm, joining efficiencies were below 90%, whereas values at 0.6–0.9 mm reached approximately 91–95% and satisfied the screening criteria. When hatching distance increased further to 1.0 and 1.1 mm, joining efficiencies decreased to approximately 87% and 84%, respectively. Pores were also observed in the overlap regions between adjacent tracks at these distances. Therefore, 0.6, 0.7, 0.8, and 0.9 mm were selected as the hatching distance levels for the subsequent orthogonal experiments.

The cross-sectional morphologies, dilution ratios, and joining efficiencies obtained from the single-factor experiments were used to select four levels for each of the four factors. These factors and levels for the subsequent Taguchi orthogonal experiments are listed in Table 3.

### 3.2. Orthogonal Experimental Results and Signal-to-Noise Ratio Analysis

Blue–near-infrared hybrid laser L-DED experiments were conducted using an L16 (4^4^) orthogonal array based on the factors and levels listed in Table 3. The experimental design and measured dilution ratios are presented in Table 4.

**Figure 12:**
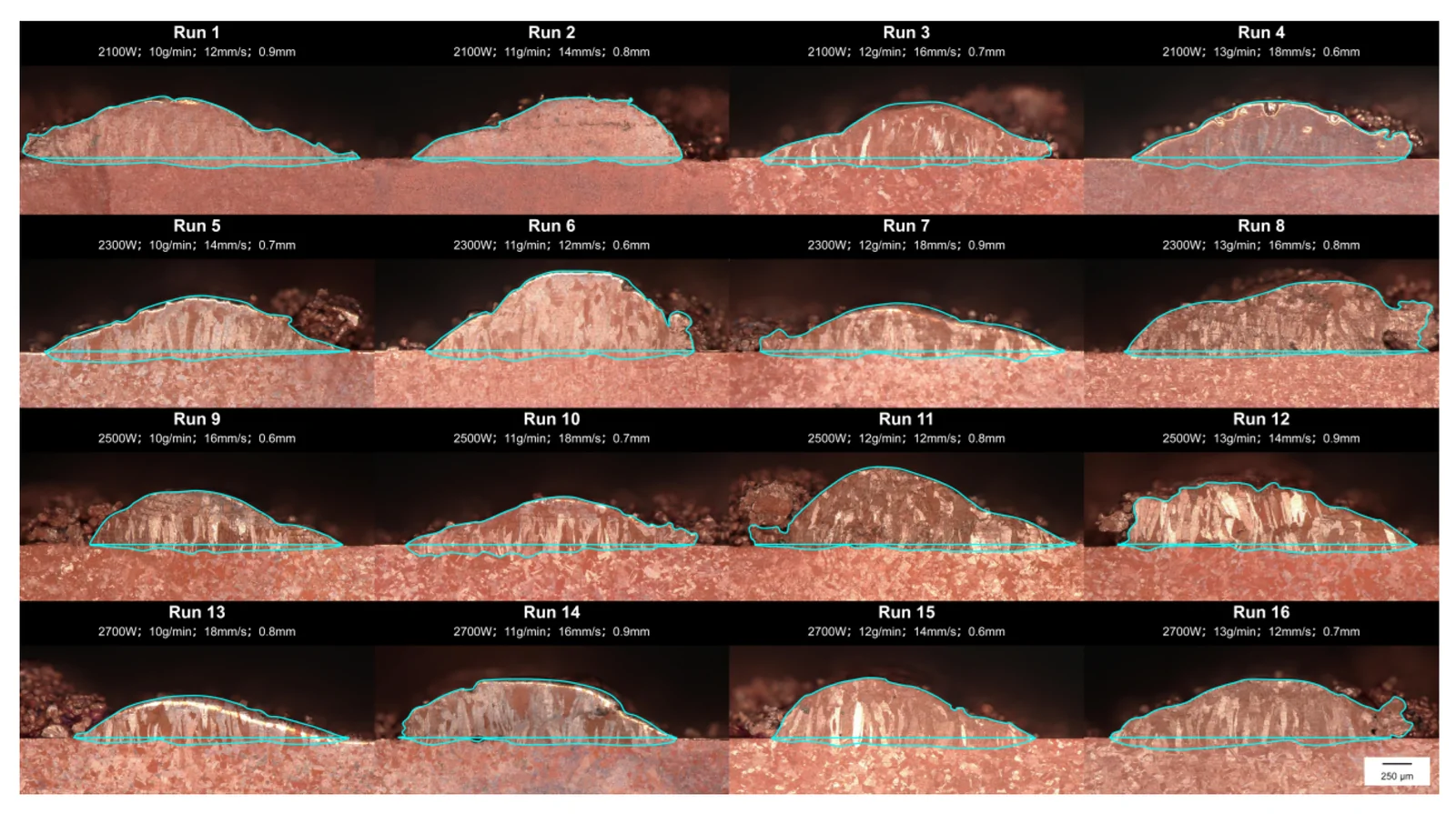
Representative cross-sectional morphologies of the deposits from each run of the L16 orthogonal experiments.

Figure 12 shows a representative cross-section from one independent track for each of the 16 orthogonal experimental runs. The labels 1–16 correspond to the run numbers in Table 4. These images allow visual comparison of the deposit cross-sectional profiles and melted substrate regions under different parameter combinations. As shown in Table 4, mean dilution ratios ranged from 7.13% to 21.04%. Run 10 had the highest mean dilution ratio of 21.04%, while run 8 had the lowest value of 7.13%. The mean dilution ratios of runs 3, 7, 13, and 15 were 14.86%, 15.41%, 15.64%, and 15.66%, respectively. Each differed from the target of 15% by less than one percentage point. However, the mean alone cannot capture both the deviation from the target and the variability among the three independent track measurements. Nominal-the-best S/N ratio analysis was therefore used to further evaluate the parameter levels.

The optimization objective was to achieve a dilution ratio close to 15%, so the orthogonal experimental results were analyzed using the nominal-the-best S/N ratio. The target-deviation-based nominal-the-best definition reported by Roy [29] was adopted. For each run, the mean squared deviation of the replicate results from the target was calculated and converted into an S/N ratio using Equation (15):

(15) $$S/N=-10\mathrm{lg}\left[\frac{1}{n}\sum_{i=1}^{n}(y_{i}-m{)}^{2}\right]$$

Here, *S*/*N* is the signal-to-noise ratio, and *n* is the number of replicates for each experimental run. *y_i_* is the dilution ratio measured in the *i*th replicate, and *m* is the target dilution ratio, set at 15% in this study. According to Equation (15), smaller deviations from the target produce a higher S/N ratio. A higher value therefore indicates that the parameter combination achieves dilution ratios closer to the target and provides better target control.

**Figure 13:**
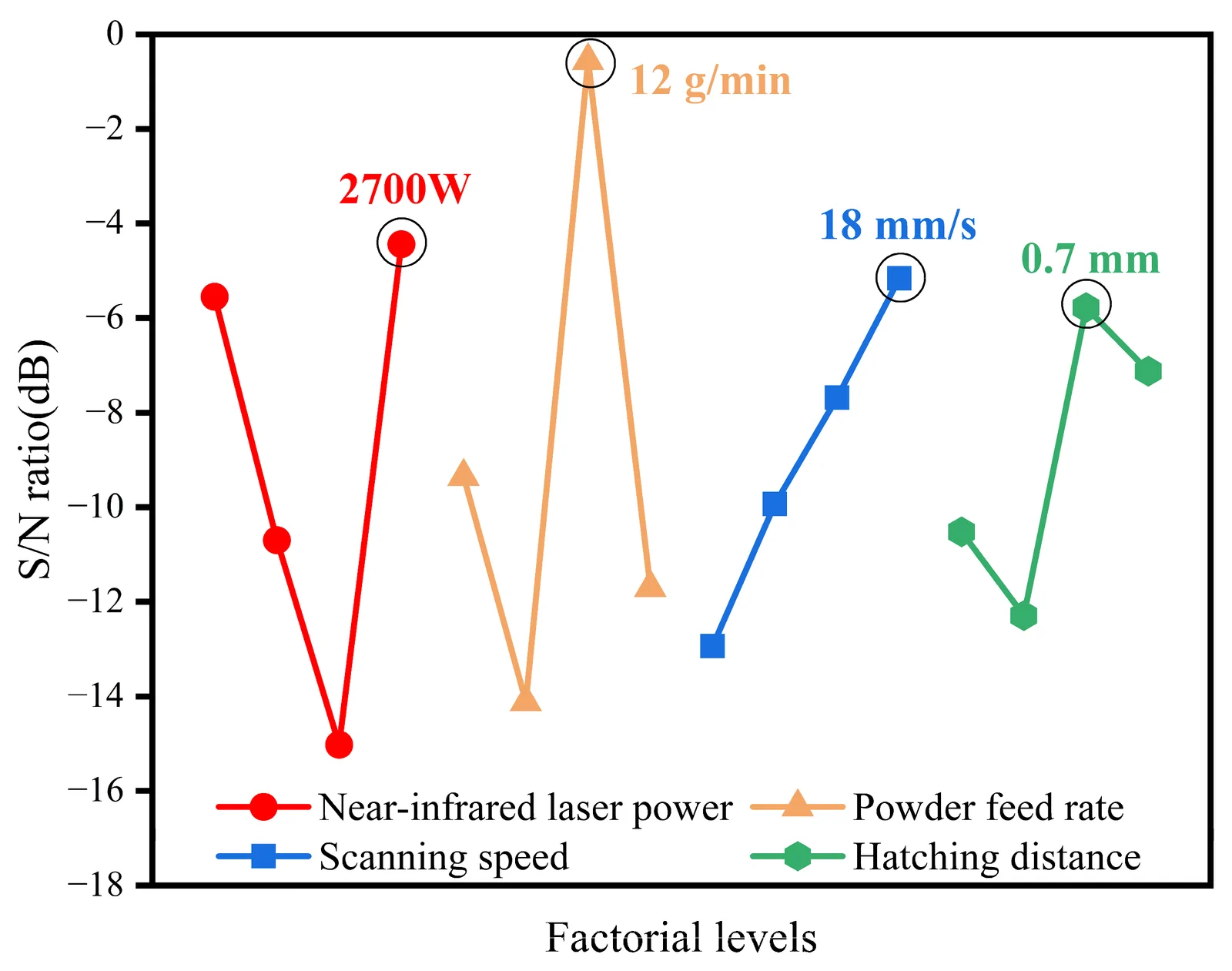
Mean *S*/*N* ratios at different levels of each factor.

The nominal-the-best S/N ratio for each run was calculated from the three independent measurements in Table 4. The mean S/N ratio at each level of each factor was then calculated, as presented in Table 5 and Figure 13. Factor A had its highest mean S/N ratio at level 4, with a value of −4.438 dB. Factor B reached its maximum at level 3, with a value of −0.555 dB. Factor C reached its maximum at level 4, with a value of −5.161 dB. Factor D reached its maximum at level 3, with a value of −5.775 dB. Based on the principle that higher S/N ratios are preferable, A4B3C4D3 was identified as the optimized parameter combination. This corresponded to a near-infrared laser power of 2700 W and a powder feed rate of 12 g/min. The scanning speed and hatching distance were 18 mm/s and 0.7 mm, respectively.

S/N ratio analysis evaluated the combined performance of dilution ratios in approaching the 15% target at different parameter levels. The optimized parameter combination was selected based on this analysis. ANOVA was then performed using the raw dilution ratio data from the three independent tracks for each run. This evaluated the statistical significance and percentage contribution of each factor’s main effect on dilution ratio variation.

### 3.3. ANOVA

ANOVA was performed using the raw dilution ratio data from the three independent tracks for each run in Table 4. The analysis evaluated the statistical significance and percentage contribution of each process parameter’s main effect on dilution ratio. The results are presented in Table 6, with the significance level set at α = 0.05.

As shown in Table 6, the *p*-values for powder feed rate, scanning speed, and hatching distance were 2.54 × 10^−5^, 1.85 × 10^−14^, and 1.05 × 10^−13^, respectively. All three values were below 0.05, indicating statistically significant effects on dilution ratio at (*α* = 0.05). Near-infrared laser power had a *p*-value of 0.063, exceeding 0.05, and its effect was not statistically significant at this level. Scanning speed, hatching distance, powder feed rate, and near-infrared laser power contributed 43.91%, 39.00%, 7.52%, and 1.78%, respectively, while the error term contributed 7.79%. Within the tested parameter ranges, the factors ranked C > D > B > A in their contributions to dilution ratio variation. This corresponded to scanning speed > hatching distance > powder feed rate > near-infrared laser power. Together with the S/N ratio results in Section 3.2, these findings supported the use of A4B3C4D3 for subsequent validation experiments.

### 3.4. Validation of the Optimized Process Parameters and Cross-Sectional Formation Results

**Figure 14:**
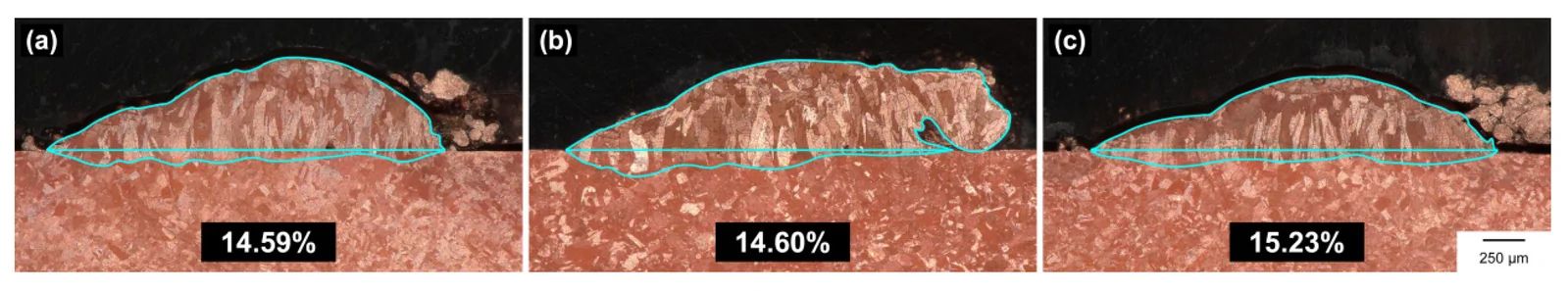
Cross-sectional morphologies of three independent tracks prepared using the optimized parameters: (**a**) 14.59%; (**b**) 14.60%; (**c**) 15.23%.

Three independent tracks were prepared using the optimized parameters: a near-infrared laser power of 2700 W and a powder feed rate of 12 g/min. The scanning speed and hatching distance were 18 mm/s and 0.7 mm, respectively. Their cross-sectional morphologies are shown in Figure 14. All three tracks formed complete deposit cross-sections, with continuous interfaces between the main body of the deposited layer and the substrate. Melted substrate regions were observed below the substrate surface in all three tracks. The corresponding dilution ratios were 14.59%, 14.60%, and 15.23%, with a mean of 14.81% and a standard deviation of 0.37 percentage points. The mean dilution ratio differed from the 15% target by 0.19 percentage points, corresponding to a relative deviation of 1.29%. Under the tested conditions, A4B3C4D3 achieved dilution ratios close to the target in three independent deposition trials, validating the Taguchi optimization results.

**Figure 15:**
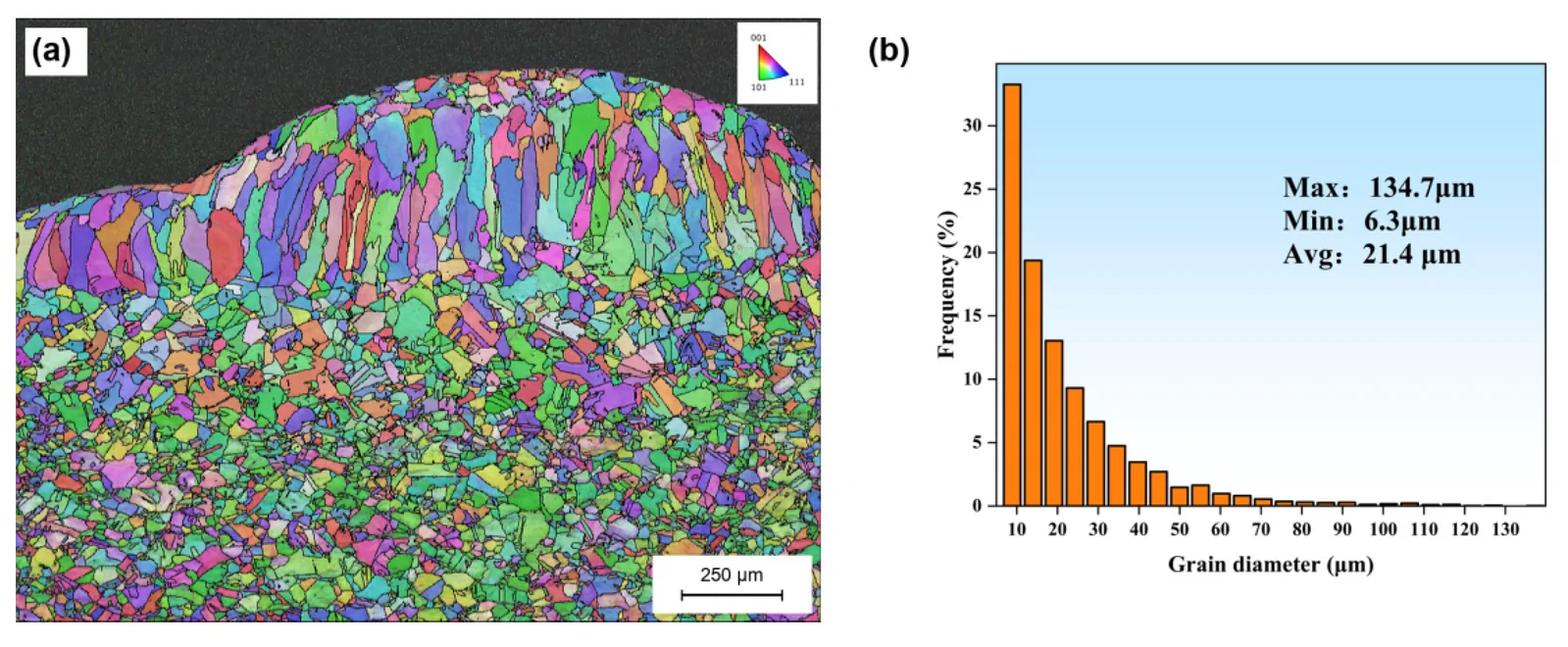
EBSD results of the CuCrZr/T2 pure copper deposited cross-section produced using the optimized parameters: (**a**) overlay of band contrast (BC), inverse pole figure (IPF), and grain boundaries; (**b**) grain equivalent circle diameter distribution.

Cross-sectional EBSD analysis was performed on the optimized single-track specimen shown in Figure 14c. The results are presented in Figure 15. As shown in Figure 15a, the substrate consisted mainly of equiaxed grains with irregular morphologies. The deposited layer interior was dominated by elongated grains extending approximately from the fusion line toward the curved deposit surface. Relatively small grains were observed near the top and both sides. Different grain colors in the IPF map indicated orientation differences between grains. Figure 15b shows the grain equivalent circle diameter distribution within the analyzed region. The minimum, maximum, and mean diameters were 6.3, 134.7, and 21.4 μm, respectively. Smaller grain sizes predominated, and their frequency generally decreased as grain size increased.

**Figure 16:**
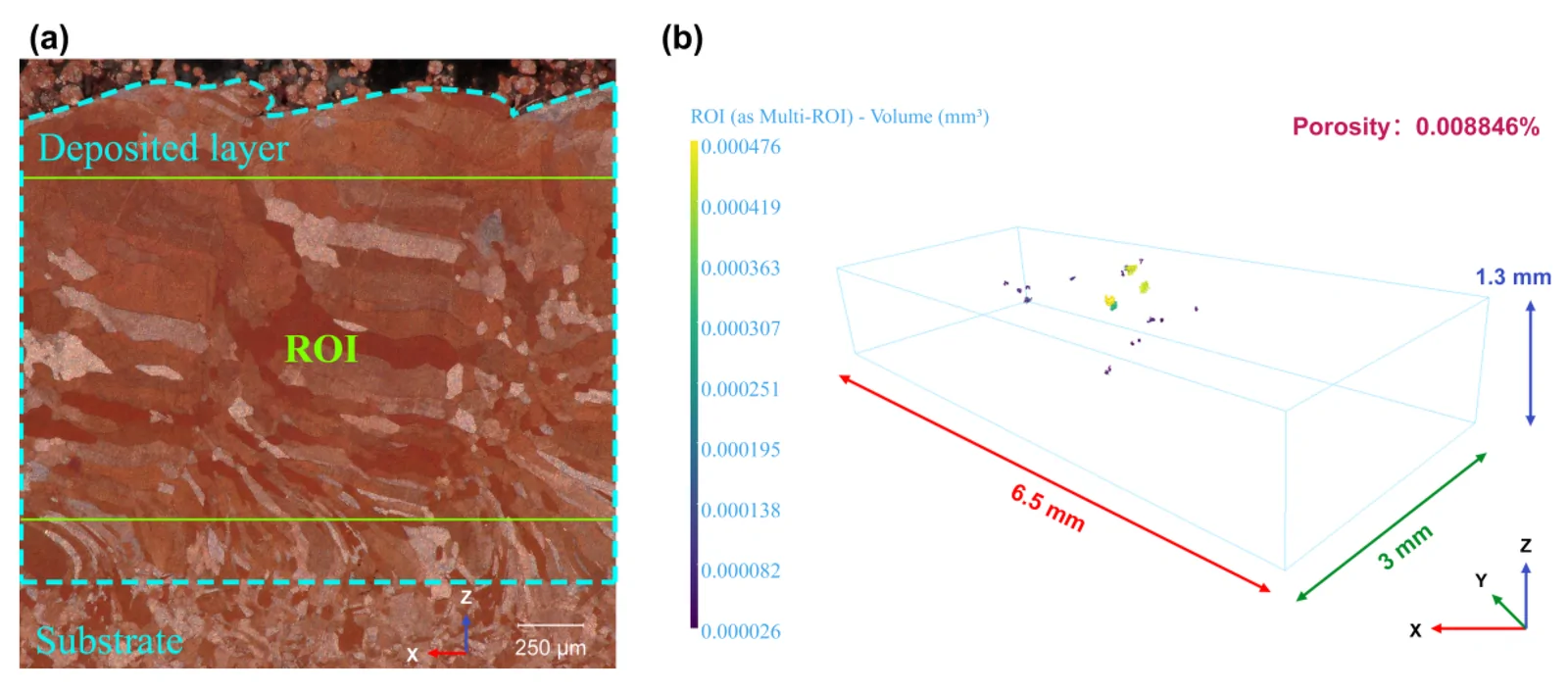
CT analysis of the multilayer, multitrack CuCrZr deposit produced using the optimized parameters: (**a**) metallographic image of the deposited region and selection of the region of interest (ROI); (**b**) three-dimensional pore distribution within the ROI, with color indicating individual pore volume.The red, green, and blue arrows in (**b**) indicate the X, Y, and Z directions, respectively, with corresponding ROI dimensions of 6.5, 3, and 1.3 mm.

Following characterization of cross-sectional formation and grain features, CT was used to evaluate internal porosity in the optimized multilayer, multitrack CuCrZr deposit. The results are shown in Figure 16. An ROI measuring 6.5 mm × 3 mm × 1.3 mm was selected within the deposited region shown in Figure 16a. This region was used for three-dimensional reconstruction and pore quantification. As shown in Figure 16b, a small number of discrete pores were observed within the ROI. Their volumes varied, but the pores were generally small. The measured volumetric porosity was 0.008846%, corresponding to a relative density of approximately 99.9912%. The optimized multilayer, multitrack deposit therefore had a low pore content within the selected ROI.

### 3.5. Microhardness and Tribological Performance

**Figure 17:**
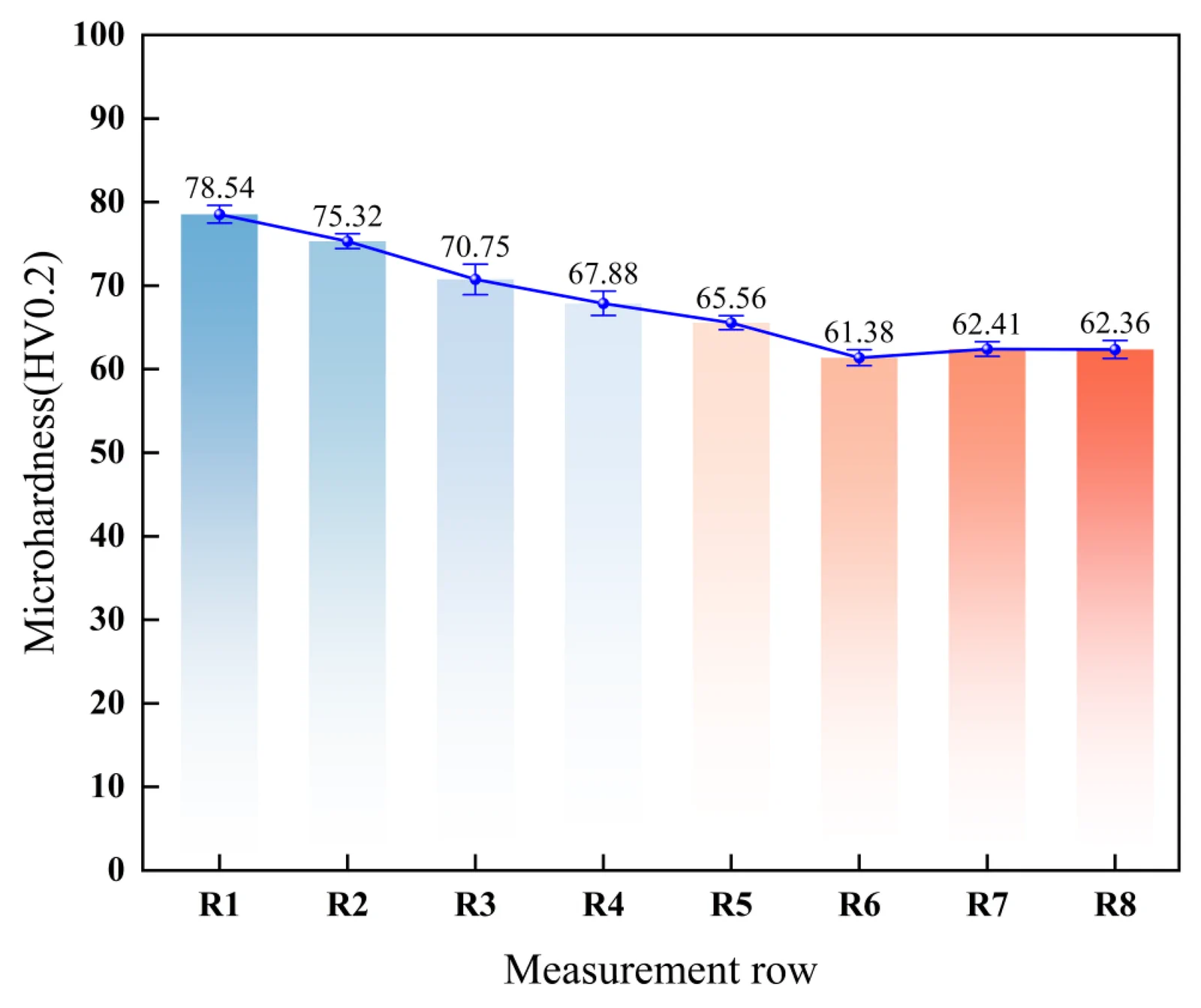
Microhardness test results.

Figure 17 shows the mean microhardness and standard deviation for each measurement row. The mean microhardness values in the deposited region were 78.54, 75.32, 70.75, and 67.88 HV0.2 for R1–R4, respectively, decreasing progressively with row number. The corresponding values in the substrate region were 65.56, 61.38, 62.41, and 62.36 HV0.2 for R5–R8, respectively. Values for R6–R8 remained relatively stable within the range of 61.38–62.41 HV0.2. The overall mean microhardness across the 16 points in the deposited region was 72.93 HV0.2. The corresponding value for the 16 points in the substrate region was 62.93 HV0.2, giving a 15.90% increase in the deposited region. Standard deviations for the four measurements in each row ranged from 0.84 to 1.82 HV0.2, indicating small within-row hardness variations. Overall, the CuCrZr deposited region had higher microhardness than the T2 pure copper substrate region.

**Figure 18:**
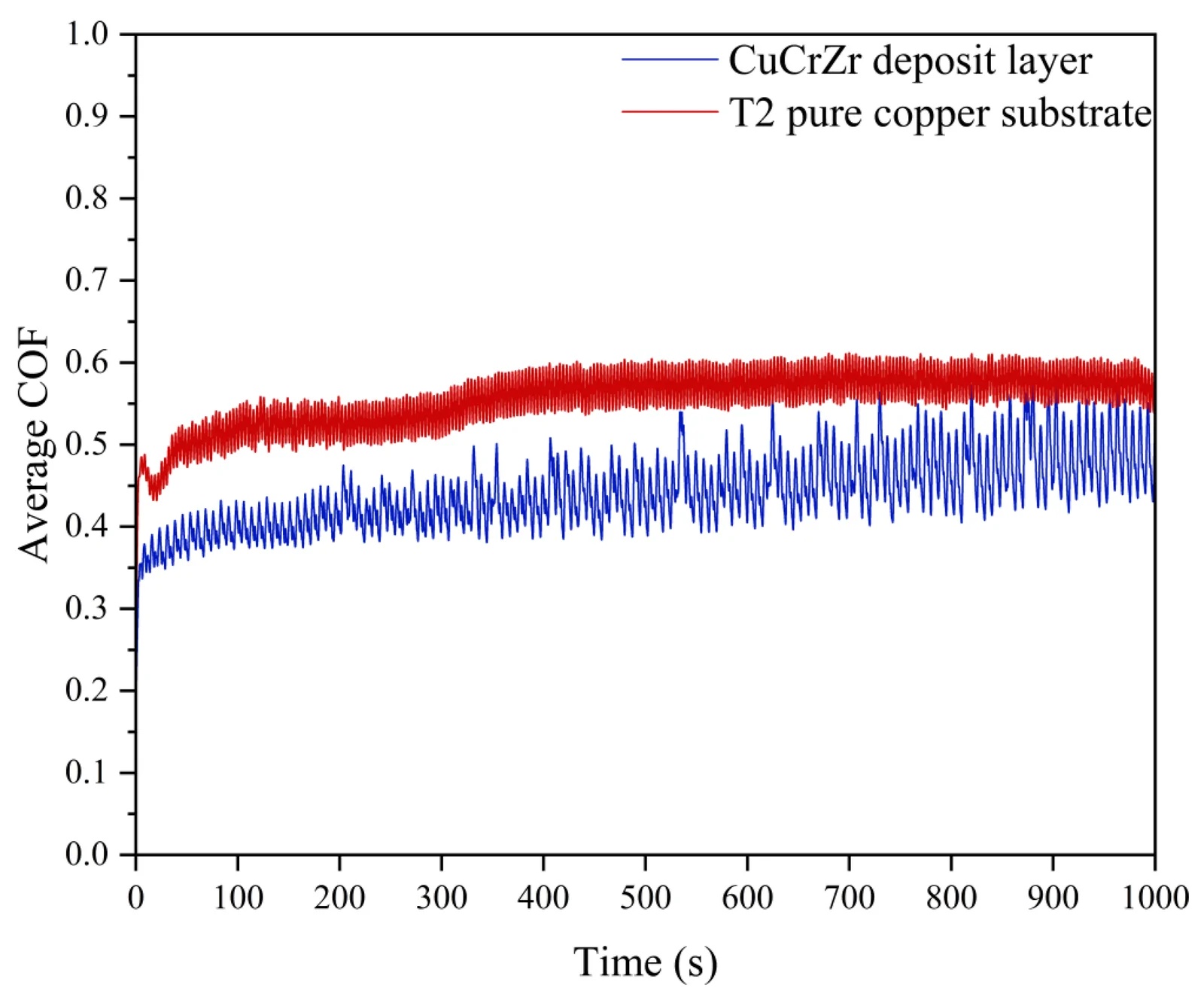
Coefficient-of-friction curves of the T2 pure copper substrate and CuCrZr deposited layer.

Figure 18 shows the mean coefficient-of-friction curves obtained from three independent tests for the T2 pure copper substrate and CuCrZr deposited layer. Both mean curves rose rapidly during the initial stage and subsequently fluctuated within a certain range. The mean coefficients of friction over the full test duration were 0.551 for the substrate and 0.438 for the deposited layer. The deposited layer therefore showed a 20.5% reduction relative to the substrate. Its mean coefficient of friction remained below that of the T2 pure copper substrate for most of the test duration.

**Figure 19:**
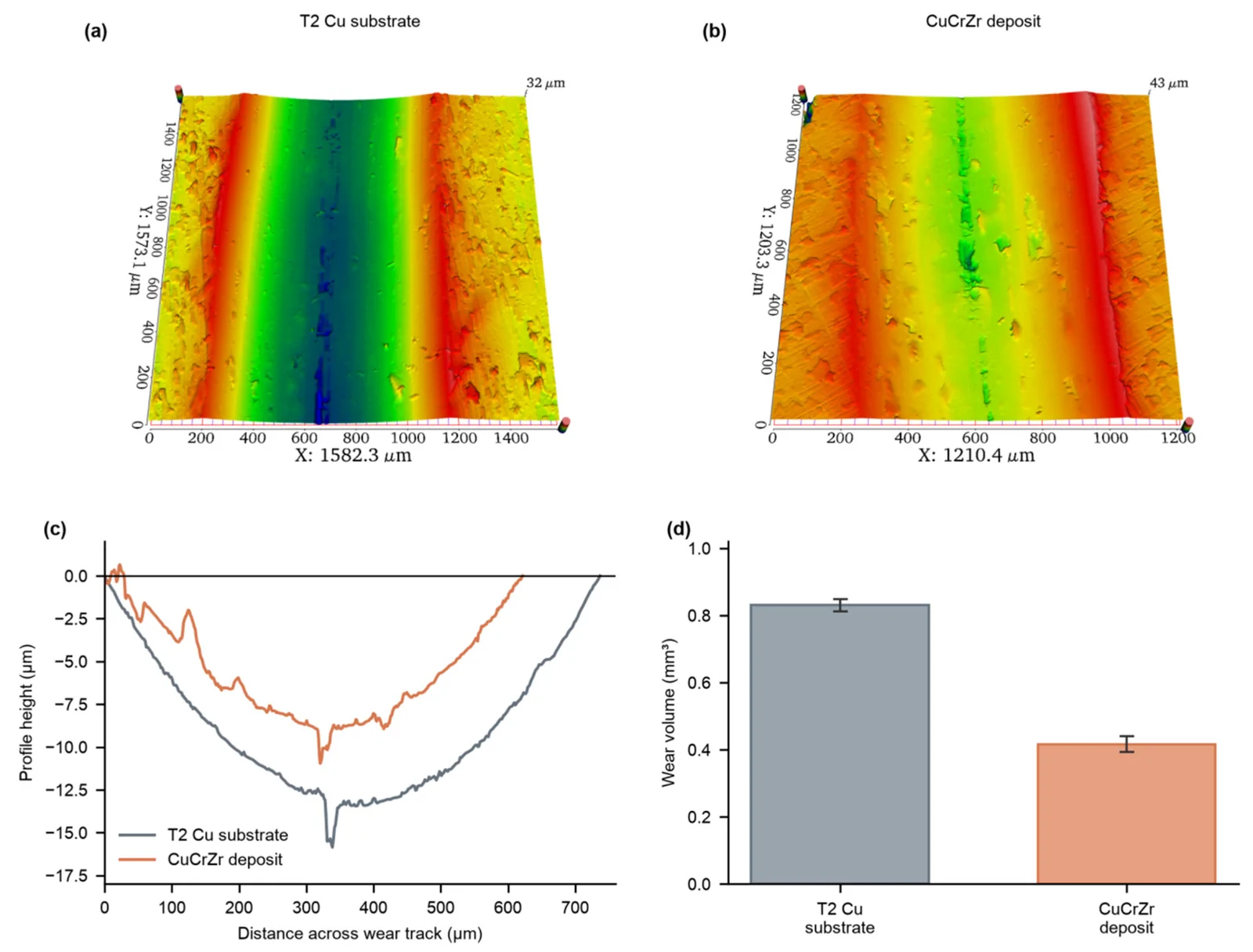
Wear track topographies and wear volumes of the T2 pure copper substrate and CuCrZr deposited layer: (**a**) 3D wear track topography of the T2 pure copper substrate; (**b**) 3D wear track topography of the CuCrZr deposited layer. In (**a**,**b**), colors indicate relative surface height, from low (blue) to high (red); (**c**) representative wear track cross-sectional profiles; (**d**) wear volume. Bars represent the means of three independent wear tracks, and error bars indicate standard deviations.

Figure 19 compares the 3D wear track topographies, representative cross-sectional profiles, and wear volumes of the T2 pure copper substrate and CuCrZr deposited layer. The representative profiles showed an overall deeper and wider wear track on the substrate, whereas the track on the deposited layer was comparatively shallower. Wear volumes were calculated separately from the cross-sectional profiles of the three independent wear tracks. The mean wear volumes were 0.831 ± 0.018 mm^3^ for the T2 pure copper substrate and 0.416 ± 0.024 mm^3^ for the CuCrZr deposited layer. The corresponding specific wear rates were 5.54 × 10^−4^ and 2.78 × 10^−4^ mm^3^/(N·m), respectively. Both wear volume and specific wear rate were 49.9% lower for the CuCrZr deposited layer than for the T2 pure copper substrate.

### 3.6. Numerical Simulation of Temperature Fields and Molten Pool Flow During Single-Track and Two-Track Deposition

**Figure 20:**
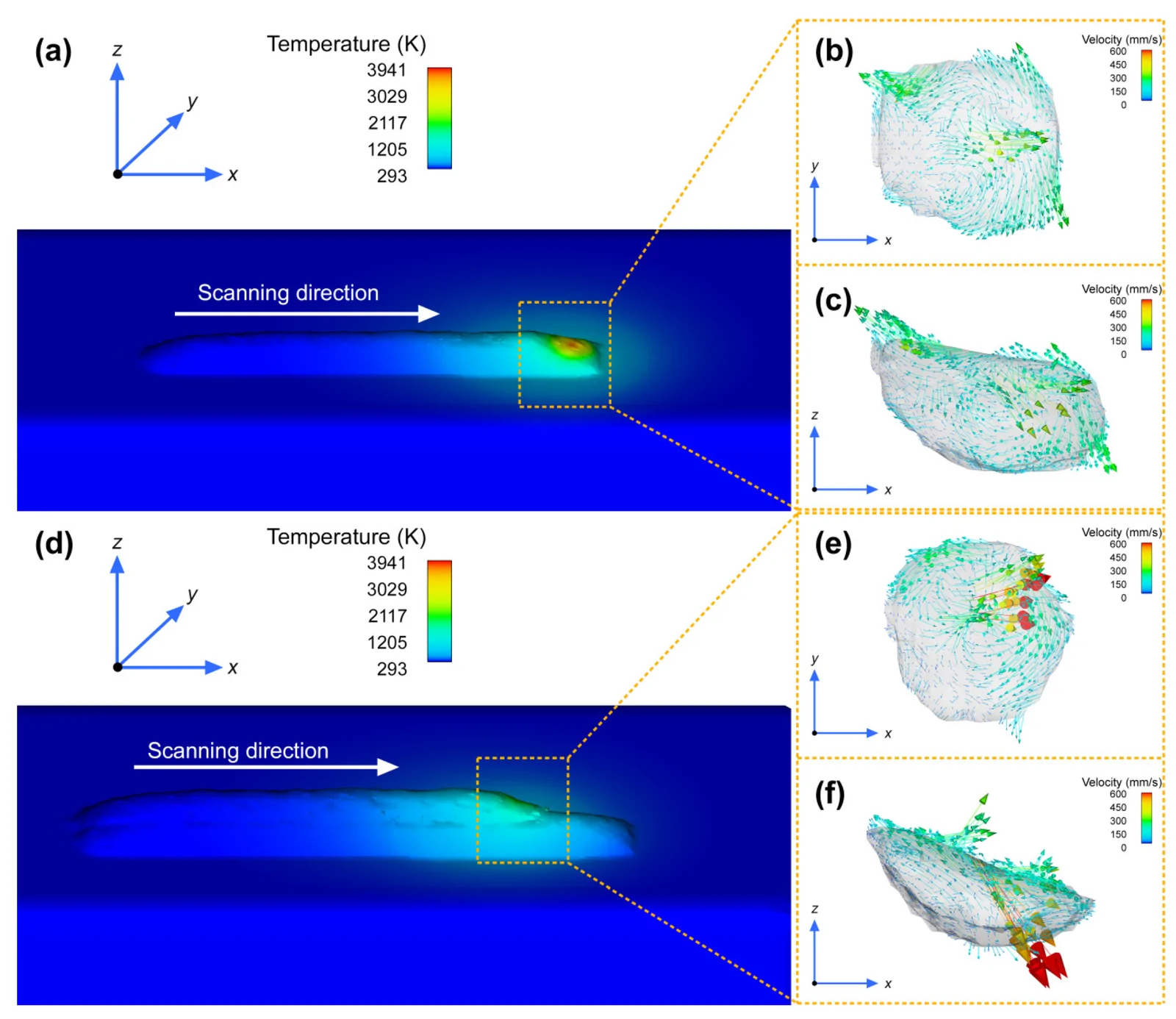
Temperature fields and molten pool flow characteristics during single-track and two-track blue–near-infrared hybrid laser deposition using the optimized parameters: (**a**) single-track temperature field; (**b**) single-track molten pool velocity field in the x-y plane; (**c**) single-track molten pool velocity field in the x-z cross-section; (**d**) two-track temperature field; (**e**) two-track molten pool velocity field in the x-y plane; (**f**) two-track molten pool velocity field in the x-z cross-section.

Figure 20 shows the temperature and molten pool velocity fields for single-track and two-track deposition using the optimized parameters. As shown in Figure 20a, the high-temperature region during single-track deposition was concentrated near the moving heat source. A thermal tail extended behind the heat source along the scanning path, and temperature decreased with distance from the heat source. During two-track deposition, the high-temperature region was also located near the current heat source. However, the surrounding temperature distribution differed from that during single-track deposition, as shown in Figure 20d.

Figure 20b,c show the single-track molten pool velocity fields in the x-y plane and x-z cross-section, respectively. The velocity vector distribution in the single-track molten pool plane was relatively symmetric. Surface flow and internal return flow formed a circulation pattern. During deposition of the second track, the velocity vector distribution shifted noticeably and became less symmetric, as shown in Figure 20e. With the same velocity color scale, more pronounced local high-velocity regions were also observed in the second-track molten pool. Its cross-sectional flow distribution differed from that of the single-track molten pool, as shown in Figure 20f.

## 4. Discussion

### 4.1. Effects of Process Parameters on Deposit Quality, Microstructure, and Performance

The optimized parameter combination identified in this study comprised a near-infrared laser power of 2700 W and a powder feed rate of 12 g/min. The scanning speed and hatching distance were 18 mm/s and 0.7 mm, respectively. This combination balances powder melting, material accumulation, substrate melting, and fusion between adjacent tracks. The changes in deposit morphology shown in Figure 8, Figure 9, Figure 10 and Figure 11 reflect the interactions among these processes. The powder feed rate and scanning speed jointly determine the material input per unit scan length. This affects the deposition height and the heat required to melt the powder. The hatching distance alters the overlap between adjacent tracks, thereby affecting local remelting and bonding. Copper’s high thermal conductivity rapidly transfers local heat into the surrounding substrate, affecting local melting, fusion, and contraction during cooling. If heat input, material input, and track overlap are poorly matched, the cross-section may exhibit pronounced bulging, irregular contours, or insufficient local bonding. Such deviations from a regular arc-shaped profile can, in turn, affect the dilution ratio. Changes in the dilution ratio therefore reflect both substrate melting and the accumulation of deposited material. Zardoshtian et al. similarly found that stable deposition during multilayer directed energy deposition of CuCrZr requires matching laser power to powder input per unit scan length. They also reported the need to maintain suitable overlap between adjacent tracks [30]. In this study, scanning speed and hatching distance contributed 43.91% and 39.00%, respectively, to the variation in dilution ratio. By comparison, near-infrared laser power contributed only 1.78% to this variation. These results indicate that material input and track overlap have a greater influence on dilution once near-infrared power is sufficient to maintain good interfacial bonding. This difference is related to copper’s low initial absorptivity for near-infrared laser radiation, which causes a large fraction of the incident energy to be reflected [1,2].

**Figure 21:**
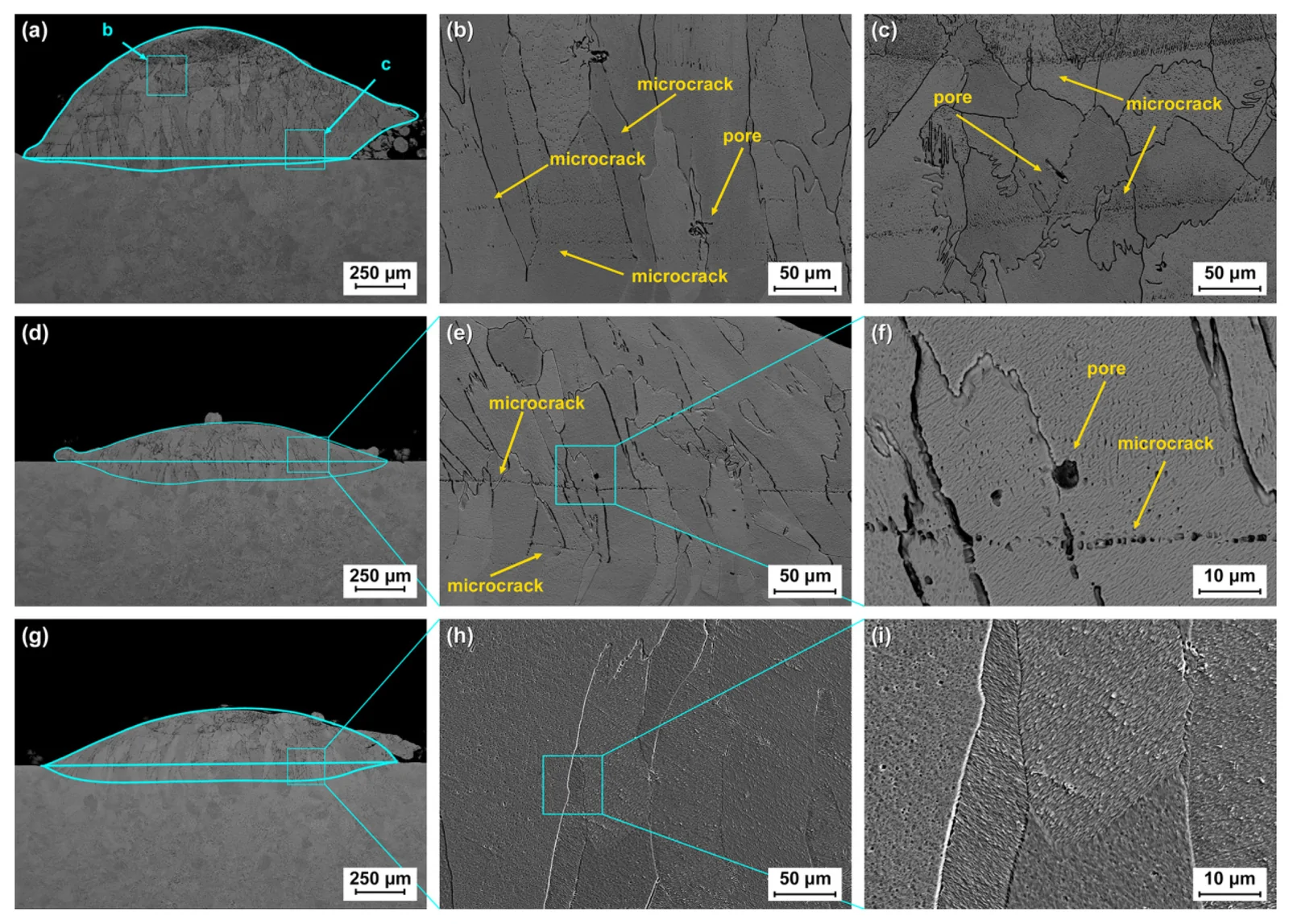
Etched cross-sectional SEM images of single-track CuCrZr deposits with different dilution ratios: (**a**–**c**) low dilution ratio, 2.34%, with (**b**) and (**c**) showing magnified views of the cyan boxes labeled b and c in (**a**), respectively; (**d**–**f**) high dilution ratio, 45.34%; (**g**–**i**) dilution ratio close to the 15% target under the optimized parameters, 15.61%.

Each row presents the overall cross-section followed by local images at two magnifications. Boxes and connecting lines indicate the regions selected for magnification. Figure 21 shows the deposited cross-sectional morphologies and local defects at different dilution ratios. In Figure 21a, the single-track specimen with a dilution ratio of 2.34% had a small melted substrate region and a markedly raised deposit. The specimen with a dilution ratio of 45.34% had a wider, flatter cross-section and a lower deposit height above the original substrate surface. This morphology is shown in Figure 21d. The magnified images revealed pores and cracks in the regions bonded to the substrate in both specimens. In the low-dilution specimen, cracks occurred at the bonded region and extended into the deposited layer, as shown in Figure 21b,c. This defect distribution can be interpreted in terms of the combined effects of local bonding conditions and cooling contraction. Copper’s high thermal conductivity rapidly transfers local heat to the surrounding substrate. The deposited region contracts during cooling, while restraint from the substrate and solidified material generates thermal stress. In L-DED of CuCr1 copper alloy and a nickel-based alloy, Khaimovich et al. mainly attributed cracking to macroscopic residual stresses arising from thermal expansion differences. They also observed crack propagation along grain boundaries. These findings indicate that thermal stress is an important factor in cracking in related copper alloy deposition systems [31]. In the present low-dilution specimen, regions with insufficient local bonding formed structurally weak locations. Pores and interfacial discontinuities also provided sites for local stress concentration. Cracks could therefore initiate more readily at these locations and propagate into neighboring deposited regions.

By contrast, the optimized single-track specimen with a dilution ratio of 15.61% formed a relatively complete curved cross-section. Its interface with the substrate was continuous, and no obvious pores or cracks were observed in the examined region. Suitable parameter matching should therefore balance substrate melting with deposited material accumulation. It should maintain complete interfacial bonding while bringing the dilution ratio close to the target. The relatively complete cross-sectional bonding under the optimized parameters was consistent with the internal quality of the multilayer, multitrack deposit. The selected ROI in Figure 16 had a relative density of approximately 99.9912%, indicating very low pore content within this examined region. Local cross-sectional SEM observations and three-dimensional CT pore measurements together indicate a relatively intact, dense deposited structure under the optimized parameters. This structure was obtained while maintaining a dilution ratio close to the target.

Within this relatively intact structure, local solidification conditions further determined grain morphology and orientation. Different grain regions in Figure 21h,i exhibited differently oriented etching patterns. The IPF map in Figure 15 also showed orientation differences between grains. Elongated grains extended from the fusion line toward the curved deposit surface. Their morphology was consistent with directional solidification associated with heat transfer through the substrate. The relatively small grains near the top and both sides reflected local differences in temperature gradient, solidification rate, and nucleation conditions. Zardoshtian et al. also observed columnar grains extending from the fusion region toward the deposited surface in CuCrZr L-DED. Finer grains occurred near the top surface, and this spatial distribution was linked to local solidification conditions [30]. The coexistence of elongated and smaller grains therefore reflects spatial variations in heat transfer and solidification conditions within the deposited cross-section. Grains with different orientations respond differently to slip under external loading. Slip transfer across grain boundaries also depends on local stress, dislocation characteristics, and grain boundary geometry. Haug et al. showed that crystal orientation and grain boundaries affected dislocation paths and slip transfer between neighboring grains beneath a copper sliding contact [32]. Their findings provide a basis for relating microstructural features to local plastic deformation. Regions with smaller grains have a greater grain boundary density. Grain boundaries constrain dislocation motion and can increase local resistance to plastic deformation. The relationship between grain size and hardness is also consistent with the relationship described by Hall [33]. Shen et al. further reported that grain and subgrain boundaries impeded dislocation motion and contributed to strengthening in CuCrZr [34]. The spatial differences in grain morphology, size, and boundaries in Figure 15 provide a microstructural explanation for the hardness variations in Figure 17. Reheating and local remelting during subsequent multilayer deposition can further alter the thermal history and microstructural state of existing deposited regions [30]. In this microstructural context, the higher microhardness of the deposited layer reflects greater local resistance to plastic deformation than that of T2 pure copper.

The relative density and resistance to plastic deformation of the deposited layer jointly affect subsequent wear behavior. Under repeated sliding against the Al_2_O_3_ counterbody, higher hardness helps reduce local indentation and shear deformation. It also suppresses material migration toward the wear track edges and the accumulation of repeated deformation. Higher relative density reduces weak locations within the contact region, helping maintain continuous local load support and limit material detachment. Shen et al. found that hardening CuCrZr produced shallower, narrower wear tracks, less material pile-up along their edges, and lower wear loss [34]. This relationship is consistent with the changes shown in Figure 17, Figure 18 and Figure 19. The deposited layer had 15.90% higher mean microhardness than the substrate and a comparatively shallower wear track. Both wear volume and specific wear rate decreased by 49.9%. The improved wear resistance under the optimized conditions can therefore be understood through structural integrity and microstructural constraints on plastic deformation. Structural integrity reduces weak locations for local damage, while the microstructure restricts deformation and material migration during sliding contact. Together, these effects reduce material loss during repeated frictional contact.

### 4.2. Temperature Field and Flow Characteristics of the Molten Pool During Blue–Near-Infrared Hybrid Laser Deposition

Under hybrid laser irradiation, blue light benefits from the higher absorptivity of copper at shorter wavelengths, providing relatively stable energy input for molten pool formation. The near-infrared laser supplies additional local heat and influences substrate melting and molten pool flow [9,12,15,35]. Previous studies found that blue light improved copper molten pool stability. Local energy input from the near-infrared laser altered temperature gradients and flow within the molten pool [15,25,36]. Based on previous studies, the circulation shown in Figure 20b,c is mainly driven by surface tension differences caused by surface temperature gradients. This driving mechanism is known as the Marangoni effect [25]. Molten metal moves outward along the free surface and returns through the molten pool interior. This circulation promotes the redistribution of heat and material within the molten pool.

Compared with single-track deposition, the velocity vector distribution in the second-track molten pool shifted noticeably because the second track overlapped the preceding track. Existing deposited material on the overlap side created local geometry and thermal history different from those on the opposite side. Reheating and local remelting of the preceding track further altered temperature gradients and melt flow in the overlap region [37]. Previous studies of multitrack laser cladding also observed asymmetric molten pool flow in overlap regions [38]. Local high-temperature regions provide the thermal conditions for powder melting and melting of the substrate surface layer. Circulation within the molten pool promotes melt spreading and heat exchange at the interface. Reheating and local remelting during two-track deposition help fusion between adjacent tracks. The temperature and flow fields in Figure 20 provide a qualitative explanation for the deposit formation and interfacial fusion of the validation specimens in Figure 14. This explanation relates to heat transfer and melt flow.

## 5. Conclusions

Blue–near-infrared hybrid laser L-DED enabled stable deposition of CuCrZr alloy on T2 pure copper. A parameter combination was obtained that balanced the target dilution ratio, deposit quality, and surface properties. The main conclusions are as follows:

(1) The process parameters had different effects on deposit formation and dilution behavior. Moderately increasing near-infrared laser power improved interfacial bonding compared with lower-power conditions, while deposit height and penetration depth changed relatively little. Increasing powder feed rate generally increased deposit height. Increasing scanning speed decreased deposit height and generally increased dilution ratio. Increasing hatching distance changed multitrack cross-sections from tall and narrow to wide and flat. Excessively large distances, however, reduced joining efficiency between adjacent tracks. Within the screened orthogonal experimental window, scanning speed and hatching distance were the main factors affecting dilution ratio. Their contributions were 43.91% and 39.00%, respectively.

(2) Taguchi analysis identified an optimized near-infrared laser power of 2700 W and powder feed rate of 12 g/min. The scanning speed and hatching distance were 18 mm/s and 0.7 mm, respectively. Validation experiments yielded a mean dilution ratio of 14.81%, with a standard deviation of 0.37 percentage points. The mean was close to the 15% target, and the deposits had complete cross-sections and continuous interfaces. The CT-examined region of the multilayer, multitrack specimen had a relative density of approximately 99.9912%. This parameter combination therefore balanced target dilution control with good deposit quality.

(3) FLOW-3D simulations under the optimized conditions showed a high-temperature region concentrated near the moving heat source and circulating molten metal. During deposition of the second track, the flow distribution shifted and became less symmetric. EBSD of the corresponding specimen showed predominantly elongated grains extending from the fusion line toward the curved deposit surface. Grains near the top and both sides were relatively small, and orientation differences were observed between grains. Under these conditions, the deposited layer had 15.90% higher mean microhardness than T2 pure copper and a comparatively shallower wear track. The mean coefficient of friction decreased by 20.5%, and both wear volume and specific wear rate decreased by 49.9%. The CuCrZr deposited layer therefore improved the surface hardness and wear resistance of T2 pure copper.

## Figures and Tables

**Table 1:** Chemical compositions of the CuCrZr alloy powder and T2 pure copper substrate (mass fraction, %).

Material	Cr	Zr	Fe	Si	O	Pb	Cu
CuCrZr powder	0.826	0.184	0.012	0.01	0.013	-	Bal.
T2 pure Cu substrate	-	-	0.003	-	-	0.002	Bal.

**Table 2:** Process parameter ranges for single-factor experiments.

No.	Near-Infrared Laser Power (W)	Powder Feed Rate (g/min)	Scanning Speed (mm/s)	Hatching Distance (mm)
1–8	1500–2900	11	14	/
9–17	2300	7–15	14	/
18–26	2300	11	6–22	/
27–35	2300	11	14	0.3–1.1

**Table 3:** Factors and levels for the orthogonal experiments.

Level	A: Near-Infrared Laser Power (W)	B: Powder Feed Rate (g/min)	C: Scanning Speed (mm/s)	D: Hatching Distance (mm)
1	2100	10	12	0.9
2	2300	11	14	0.8
3	2500	12	16	0.7
4	2700	13	18	0.6

**Table 4:** Results of the L16 orthogonal experiments.

No.	A (W)	B (g/min)	C (mm/s)	D (mm)	Track 1 (%)	Track 2 (%)	Track 3 (%)	Mean (%)
1	2100	10	12	0.9	10.76	8.22	11.83	10.27
2	2100	11	14	0.8	8.35	9.55	9.29	9.06
3	2100	12	16	0.7	14.87	14.59	15.13	14.86
4	2100	13	18	0.6	17.76	14.48	15.85	16.03
5	2300	10	14	0.7	19.84	17.15	17.83	18.27
6	2300	11	12	0.6	11.54	10.55	9.06	10.38
7	2300	12	18	0.9	14.71	14.72	16.80	15.41
8	2300	13	16	0.8	6.69	6.76	7.93	7.13
9	2500	10	16	0.6	9.49	11.08	11.65	10.74
10	2500	11	18	0.7	21.79	20.81	20.51	21.04
11	2500	12	12	0.8	8.83	8.63	8.95	8.80
12	2500	13	14	0.9	9.08	8.80	8.64	8.84
13	2700	10	18	0.8	16.68	14.94	15.29	15.64
14	2700	11	16	0.9	10.04	11.75	11.80	11.20
15	2700	12	14	0.6	15.84	15.15	16.00	15.66
16	2700	13	12	0.7	12.53	12.54	12.02	12.36

**Table 5:** Mean *S*/*N* ratios at different levels of each factor (dB).

Level	A	B	C	D
1	−5.549	−9.345	−12.931	−10.518
2	−10.697	−14.112	−9.931	−12.290
3	−15.021	−0.555	−7.681	−5.775
4	−4.438	−11.694	−5.161	−7.122

**Table 6:** ANOVA results.

Source	DF	Adj SS	Adj MS	F	p	Contribution
A	3	12.99	4.33	2.67	0.063	1.78%
B	3	54.89	18.30	11.26	2.54 × 10^−5^	7.52%
C	3	320.40	106.80	65.74	1.85 × 10^−14^	43.91%
D	3	284.58	94.86	58.39	1.05 × 10^−13^	39.00%
Error	35	56.86	1.63			7.79%
Total	47	729.72				

## Data Availability

The data supporting this study are presented in the article. Requests for further information may be addressed to the corresponding author.
